# Taming dimensionality in big neuroimaging data: Efficient orthonormal projective NMF via stochastic learning and data compression

**DOI:** 10.1162/IMAG.a.1355

**Published:** 2026-09-08

**Authors:** Abdalla Bani, Sung Min Ha, Thomas Earnest, Braden Yang, Pan Xiao, John Lee, Janine Bijsterbosch, Aristeidis Sotiras

**Affiliations:** Mallinckrodt Institute of Radiology, Washington University in St. Louis, St. Louis, MO, United States; Institute for Informatics, Data Science & Biostatistics (*I*^2^*DB*), Washington University in St. Louis, St. Louis, MO, United States; Department of Radiology, Perelman School of Medicine, University of Pennsylvania, Philadelphia, PA, United States; Center for AI and Data Science for Integrated Diagnostics (AI2D), Perelman School of Medicine, University of Pennsylvania, Philadelphia, PA, United States

**Keywords:** MRI, big data, NMF, data compression and stochastic optimization

## Abstract

Large-scale neuroimaging datasets present remarkable opportunities for advancing our understanding of human brain structure and function. Data-driven pattern analysis methods such as Orthonormal Projective Non-negative Matrix Factorization (opNMF) are particularly well suited to uncover multivariate relationships within these data, offering greater interpretability and reproducibility than more conventional approaches such as principal component analysis (PCA) and independent component analysis (ICA). Despite its utility in clinical computational neuroscience, the application of opNMF in large cohort studies has been impeded by computational challenges and scalability limitations. In this work, we address these issues by introducing a stochastic optimization strategy that processes mini-batches of the data, substantially improving scalability. We further accelerate computation through random data compression and leverage repulsive point processes to diversify mini-batches, reducing redundancy and the variance of updates. We first evaluated our method on gray matter tissue density maps from 1,000 participants in the Open Access Series of Imaging Studies (OASIS). Compared with the original approach, it achieved similar approximation accuracy and factor interpretability while greatly reducing computational cost. To demonstrate practical utility, we then applied the framework to 10,000 participants from the UK Biobank, identifying 20 patterns of structural covariance (PSCs) and examined associations between visceral adipose tissue (VAT) and PSC loadings, finding significant relationships for 13 PSCs in females and 11 PSCs in males, most of which were negative. We further show how these patterns refine in higher-rank decompositions with 40 and 60 components. This enhanced opNMF framework opens new possibilities for large-scale neuroimaging analyses, facilitating deeper insights into brain structure in both health and disease.

## 1 Introduction

Advancements in imaging technology and streamlined acquisition workflows have spurred the emergence of numerous large-scale neuroimaging studies of heterogeneous populations. This shift has driven neuroimaging initiatives to broadly supply high-resolution data for thousands of subjects (Glasser et al., 2013; LaMontagne et al., 2019; Sudlow et al., 2015), placing the field firmly within the domain of “Big Data.” The accessibility of these datasets presents opportunities for conducting big-data neuroimaging analyses, with greater accuracy and statistical power. This, in turn, holds the promise of advancing our understanding of brain structure in health and disease.

Developing data-driven, hypothesis-free analysis tools with a focus on large-scale studies can complement traditional methods for assessing brain structure, such as anatomical atlases based on regions of interest (ROIs). Unsupervised machine learning techniques such as principal component analysis (PCA) and independent component analysis (ICA) have been commonly employed in neuroimaging studies to derive data-driven representations of brain structure and function. However, these methods rely on complex interactions between components with opposite signs, leading to representations that are difficult to interpret, lack clear regional specificity, and often fail to generalize well to new, unseen datasets (Sotiras et al., 2015). In contrast, non-negative matrix factorization (NMF) (Lee & Seung, 1999) offers an alternative approach by factorizing the data under a non-negativity constraint. This constraint leads to a parts-based representation, where parts combine additively to form a cohesive whole.

A variant of the vanilla NMF, known as orthonormal projective NMF (opNMF) (Z. Yang & Oja, 2010), has demonstrated superior interpretability and reproducibility than principal component analysis (PCA) and independent component analysis (ICA) in structural brain analyses (Sotiras et al., 2015, 2017), supporting its increasing use in neuroimaging research. In particular, prior work showed that opNMF yields more localized, additive, and neuroanatomically meaningful components, whereas PCA and ICA tend to produce spatially diffuse patterns with mixed-sign weights (Sotiras et al., 2015). The orthogonality constraint in opNMF further identifies spatially distinct structural networks that are more stable and reproducible across initializations and cohorts (Sotiras et al., 2015). These properties make opNMF especially attractive for neuroimaging studies in which interpretability, regional specificity, and reproducibility are central goals.

For example, studies have applied these methods across multiple imaging modalities, including T1- and T2-weighted MRI (Kaczkurkin et al., 2019; Nazeri et al., 2022; Sotiras et al., 2015, 2017), fluid-attenuated inversion recovery (FLAIR) imaging (Habes et al., 2018), diffusion MRI (Bagautdinova et al., 2023; Nazeri et al., 2025; Ouyang et al., 2019), flortaucipir PET imaging (Earnest et al., 2024), functional data (Fürböck, 2020; Ghanbari et al., 2017), cortical thickness covariance analyses (Jirsaraie et al., 2019), and multimodal imaging frameworks (Ochi et al., 2022; Patel et al., 2020; Robert et al., 2022). These applications span diverse populations across the lifespan, including neonates (Nazeri et al., 2022; Ouyang et al., 2019), adolescents and young adults (Jirsaraie et al., 2019; Sotiras et al., 2017), adults (Patel et al., 2020; Robert et al., 2022), and clinical populations with neuropsychiatric and neurodegenerative conditions (Habes et al., 2018; Kaczkurkin et al., 2019; Shui et al., 2024; Varikuti et al., 2018; J. Yang et al., 2024).

Importantly, these applications have demonstrated that opNMF-derived representations capture biologically, psychologically, and clinically meaningful variation. In developmental studies, opNMF has identified structural networks aligned with functional systems and evolutionary cortical organization (Sotiras et al., 2017), coordinated patterns of preterm cortical maturation (Ouyang et al., 2019), and reproducible patterns of early postnatal white matter maturation whose developmental trajectories corresponded with microstructural indices and histological features of brain reorganization (Nazeri et al., 2022). In adolescents, NMF-derived structural covariance networks also revealed that greater impulsive choice was associated with reduced cortical thickness in networks encompassing the ventromedial prefrontal and orbitofrontal cortices, temporal pole, and temporoparietal junction, and that these networks predicted delay discounting beyond cognitive performance (Pehlivanova et al., 2018). In multimodal analyses, opNMF has revealed hippocampal microstructural organization associated with demographic and behavioral variation (Patel et al., 2020), as well as striatal microstructural components whose interindividual variability was primarily related to age and sex and further characterized through functional annotation (Robert et al., 2022).

Clinical applications have identified regional white matter lesion patterns associated with vascular risk and cognition (Habes et al., 2018), cortical thickness covariance networks associated with irritability and accelerated thinning in frontal and temporal regions involved in emotion regulation (Jirsaraie et al., 2019), and widespread cortical network disruptions associated with posttraumatic stress disorder diagnosis and symptom severity, particularly within prefrontal, cingulate, insular, motor, and sensory-processing regions (J. Yang et al., 2024). In aging and neurodegenerative disease, NMF-derived gray matter representations have supported age prediction across healthy and clinical populations, demonstrated transferability across datasets, and provided localized components that facilitate interpretation of brain-aging patterns (Varikuti et al., 2018). opNMF has also identified Alzheimer’s disease-related atrophy patterns linked to cerebrospinal fluid biomarkers and longitudinal cognitive decline (Shui et al., 2024), as well as reproducible tau covariance patterns in flortaucipir PET data that overlapped with Alzheimer’s disease-related gene-expression maps and were associated with dementia status, neuropsychological performance, and amyloid burden (Earnest et al., 2024). Collectively, these findings establish opNMF as a robust and well-validated framework for deriving biologically, psychologically, and clinically meaningful representations.

Large-scale neuroimaging has also motivated scalable adaptations of methods other than opNMF, particularly in the fMRI literature. For example, Multivariate Exploratory Linear Optimized Decomposition into Independent Components’ (MELODIC) Incremental Group-PCA (MIGP) (Smith et al., 2014) was developed to approximate group PCA for very large fMRI datasets by updating a low-rank PCA representation incrementally across subjects, thereby reducing the memory burden of full temporal concatenation. Multi-power iteration PCA (MPOWIT) (Rachakonda et al., 2016) addresses a similar scalability problem from a different angle, using memory-efficient subspace iteration to estimate the dominant PCA space for large group-ICA workflows. In contrast, neighborhood preserving embedding (NPE) (Zhao et al., 2022) methods approach dimensionality reduction as a nonlinear manifold-learning problem, seeking to preserve local neighborhood structure in the reduced representation rather than recover a PCA subspace. These methods illustrate that scalable neuroimaging analysis can be achieved through several distinct strategies, including incremental PCA, iterative subspace estimation, and manifold-based embedding. However, they differ fundamentally from opNMF. Both MIGP and MPOWIT are designed to scale the PCA-based reduction and, therefore, optimize for maximal variance, yielding mixed-signed, spatially overlapping components that can limit interpretability. In contrast, opNMF enforces a non-negativity constraint to produce a “parts-based” representation, ensuring that the resulting structural networks are purely additive and spatially localized.

Stochastic versions of opNMF, which process mini-batches of data, offer a potential solution for managing large datasets by reducing memory demands (Wen et al., 2023; Z. Yang et al., 2012). However, these variants often lack theoretical convergence guarantees and typically rely on random sampling, which can introduce redundancy and bias due to non-representative mini-batches (LeWinn et al., 2017; Sankar et al., 2016). Another computational strategy involves data compression, which accelerates updates in traditional NMF by projecting the data onto a lower-dimensional subspace that retains most of the critical information (Tepper & Sapiro, 2016). To identify this subspace, compression methods utilize structured random projections (Halko et al., 2011). The compression procedure entails obtaining the QR decomposition of a matrix product. In large-scale data settings, the matrix product becomes too extensive to fit into memory, necessitating advanced parallel computing architecture to execute the QR decomposition (tall-and-skinny-QR [TSQR]) (Tepper & Sapiro, 2016).

**Fig. 1.**
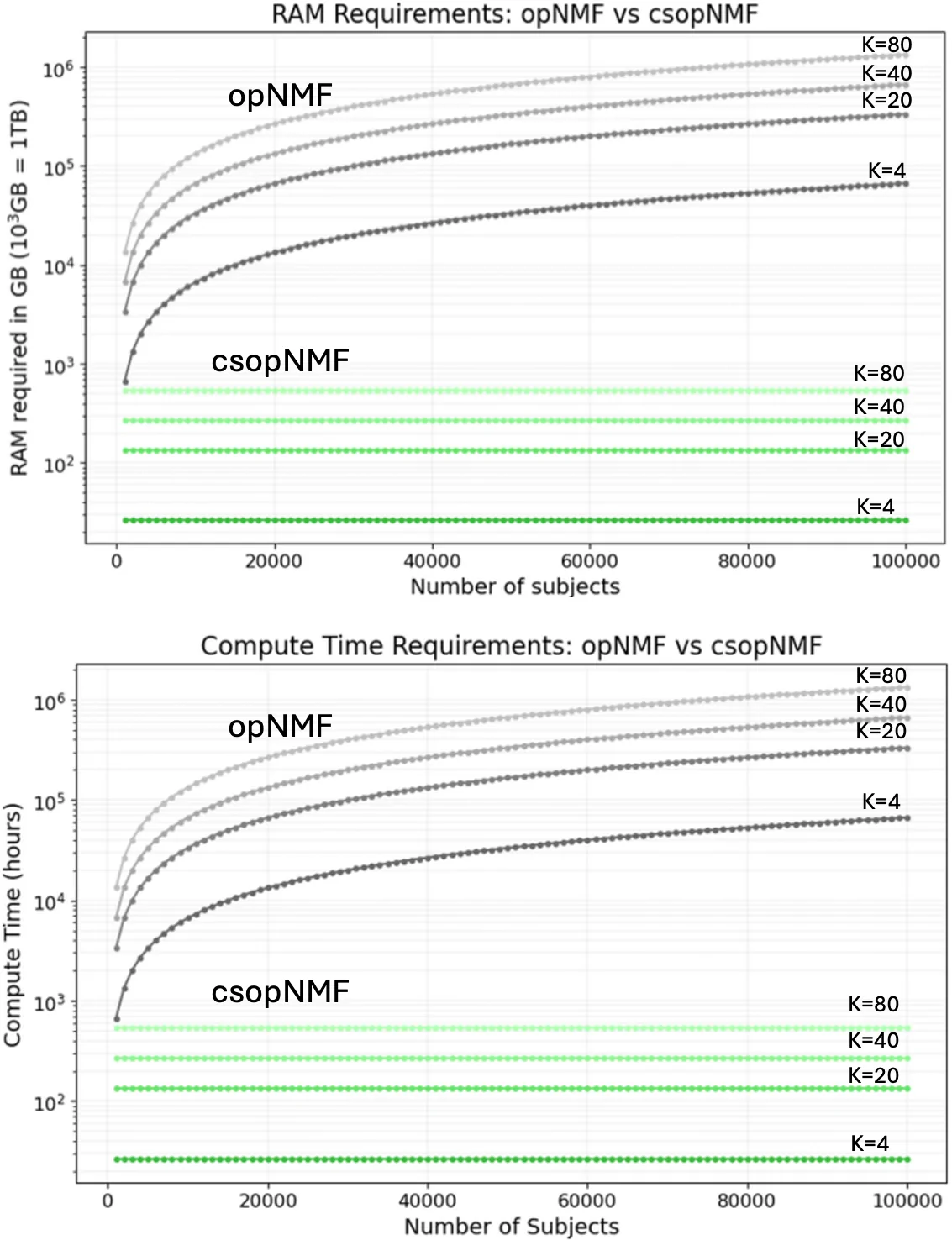
Theoretical RAM and compute-time scaling for opNMF and csopNMF across decomposition ranks k and increasing sample size. Here, k denotes the factorization rank, that is, the number of components to be estimated. Curves were generated assuming 1,200,000 voxels per subject, consistent with volumetric data registered to MNI-152 space, and subject counts ranging from 1,000 to 100,000. For csopNMF, calculations assume a fixed compressed mini-batch size of 100, so the matrix processed at each update remains constant as the total dataset size grows. Consequently, the per-update memory and computational requirements of csopNMF are approximately independent of the total number of subjects, whereas those of opNMF increase with the full sample size. Both panels are shown on a logarithmic scale to facilitate comparison across the wide range of values.

To address the above limitations and facilitate the application of opNMF in large-scale studies, we propose an algorithm specifically designed to scale efficiently to big neuroimaging datasets. Building on prior algorithmic solutions for stochastic and compressed NMF, our approach integrates the advantages of both paradigms into a unified framework. The algorithm adopts a stochastic learning strategy that performs opNMF updates on compressed mini-batches of data. We refer to this method as compressed stochastic opNMF (csopNMF). The stochastic component alleviates memory constraints by processing data in small batches, while the compression accelerates updates, thereby reducing run-time requirements (Fig. 1). To further enhance robustness, we introduce an alternative sampling strategy within the stochastic learning framework. Specifically, we incorporate repulsive sampling based on determinantal point processes (DPPs) (Kulesza & Taskar, 2012), which encourage diversity among selected samples and yield mini-batches that better represent the population. DPP sampling offers two key advantages over random sampling: (1) it can incorporate covariate information (e.g., age, sex, pathology status) to promote balanced, representative sampling and mitigate bias (Bani et al., 2023; Zhang et al., 2017) and (2) by discouraging redundant sample selection, it reduces the variance of gradient updates, enabling faster and more stable convergence. Finally, our framework provides a practical solution to the big-data QR decomposition bottleneck encountered in traditional compression algorithms. Because the method operates stochastically on mini-batches, QR decomposition is performed on smaller data subsets—eliminating the need for complex distributed computing architectures such as tall-and-skinny QR (TSQR).

Population-scale neuroimaging enables multivariate mapping of brain organization, and the UK Biobank (UKB) provides an ideal setting to do so because of its large imaging cohort with harmonized acquisition and deep phenotyping. In this work, we used UKB as a testbed to move beyond region-wise summaries and localize population patterns with csopNMF and investigate their hierarchical organization in higher-order models. We then ask how those patterns relate to visceral adiposity. A growing literature links adiposity to brain structure. In a population sample, higher total body fat was associated with smaller gray matter volume, alterations in white matter microstructure, and subcortical differences, underscoring a brain–body connection observable at scale (Dekkers et al., 2019). More recently, a midlife cohort study (N=10,001) showed that both visceral and subcutaneous abdominal fat predicted lower brain volumes, with widespread lobar associations and indications of sex differences; these effects were observed even after normalization to intracranial volume (Raji et al., 2024). However, prior analyses of adiposity and brain structure have largely relied on region- or lobe-based reporting schemes. Such predefined anatomical units can obscure distributed and overlapping effects, impose arbitrary boundaries, and fail to capture coordinated structural variation across the brain. Here, we extend adiposity–brain analyses in the UK Biobank using data-driven csopNMF-derived patterns of structural covariance. By testing pattern loadings against visceral adipose tissue, we provide an anatomically interpretable, population-scale map of VAT–brain relationships that complements and overcomes key limitations of region- and lobe-based approaches.

Note that a preliminary version of this work was presented in Bani et al. (2023). The present work extends the previous publication in multiple ways:

1. We proposed an efficient and scalable opNMF variant called csopNMF, which leverages random compression and stochastic learning in a unified framework to overcome opNMF’s computational challenges.
2. We enhanced csopNMF by incorporating a diverse sampling approach to reduce update variances.
3. We conducted an extensive performance comparison of csopNMF with both stochastic opNMF (sopNMF) and standard opNMF as baselines. Our experiments spanned two different batch sizes and three different compression scenarios providing a broad evaluation of the algorithms’ performance.
4. We further extended the evaluation by thoroughly assessing not only the computational accuracy but also the computational time and memory.
5. Furthermore, we applied csopNMF factors in a typical downstream machine learning task (age prediction) and analyzed its performance across various setups against opNMF and sopNMF. The results demonstrated that our proposed method achieved comparable accuracy with the baseline opNMF while significantly reducing computational cost.
6. We demonstrated population-scale utility by applying csopNMF to 10,000 UK Biobank participants to derive 20 components, referred to as patterns of structural covariance (PSCs), and testing brain–body links via associations between PSC loadings and VAT.
7. Finally, we extended the analysis to higher model orders of 40 and 60 components to illustrate how broader structural covariance patterns resolve into finer-grained organization.

The remainder of this paper is structured as follows. In section 2, we define the csopNMF framework, beginning with opNMF and introducing its stochastic and compressed variants. This section also describes the datasets used and outlines the validation and application experiments along with their parameter settings. In section 3, we present the findings from both the validation and application analyses. In section 4, we summarize the main results, limitations, and conclusions of our work.

## 2 Materials and Methods

### 2.1 Proposed methodology

**Fig. 2.**
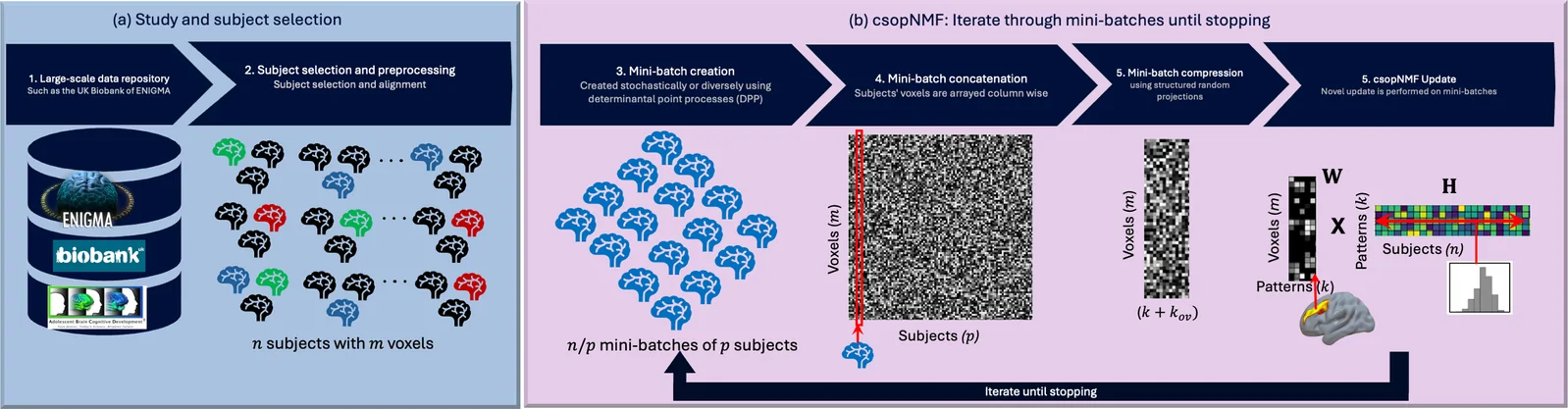
Overview of the proposed approach. (a) (1) A large-scale neuroimaging study of interest is selected for analysis. (2) Subjects meeting the inclusion criteria are identified, and their T1-weighted (T1w) images are processed using standard neuroimaging tools. (b) (3) Mini-batches are selected from the full sample either stochastically or using determinantal point processes (DPP) to promote diversity. (4) Images within each batch are vectorized and arrayed column wise to form mini-batch data matrices. (5) Mini-batches are compressed using structured random projections. (6) csopNMF is performed on compressed mini-batches, and model updates are accumulated across batches.

In this section, we first provide a brief overview of opNMF and outline the key challenges in its optimization. Then, we describe our proposed solution, including the stochastic optimization scheme, mini-batch diversification approach, and compression framework. An outline of the proposed approach is shown in Figure 2.

#### 2.1.1 Orthonormal projective non-negative matrix factorization (opNMF)

Let ${\text{x}}_{1},{\text{x}}_{2},...,{\text{x}}_{n}\in {\mathbb{R}}_{+}^{m}$ be vectorized images of n different subjects (or samples) acquired through medical imaging and m be the number of non-negative features (voxels) of each image. The data matrix X is then constructed by arraying these images column wise to obtain $\text{X}\in {\mathbb{R}}_{+}^{m\times n}$ , equivalently $\text{X}=\left[{\text{x}}_{1},{\text{x}}_{2},...,{\text{x}}_{n}\right]$, ${\text{x}}_{i}\;\in {\mathbb{R}}_{+}^{m}$. Our goal is to decompose the data matrix X into a user-specified number of components k that group features that covary consistently across the subjects. Toward this goal, we aim to solve the orthogonal non-negative matrix factorization problem (Yuan & Oja, 2005):

$$ℒ=\underset{\text{W}\geq 0}{\text{min}}{\left\|X-\text{W}{\text{W}}^{T}X\right\|}_{F}^{2}\text{ s.t.}\;\;\;{\text{W}}^{T}\text{W}=I,$$ (1)

where ℒ is the loss, $\text{W}\;\in {\mathbb{R}}_{+}^{m\times k}$ is the components matrix, $∥\cdot {∥}_{F}$ is the Frobenius norm, and I denotes the identity matrix. The previous minimization problem can be solved using the iterative multiplicative update (MU) rule (Z. Yang & Oja, 2010; Yuan & Oja, 2005)

$${\text{W}}_{t+1}\leftarrow {\text{W}}_{t}\frac{{\left(\text{X}{\text{X}}^{T}\text{W}\right)}_{t}}{{\left(\text{W}{\text{W}}^{T}\text{X}{\text{X}}^{T}\text{W}\right)}_{t}}.$$ (2)

In contrast to the traditional NMF approach (Lee & Seung, 1999), which decomposes the data matrix X into non-negatively constrained components W and coefficients H ($\text{min}{\left\|\text{X}-\text{WH}\right\|}_{F}^{2}$), opNMF takes a different route by estimating the loading coefficients $\text{H}\;\in {\mathbb{R}}_{+}^{k\times n}$ as a projection of the data onto approximated orthogonal components $\text{H}={\text{W}}^{T}\text{X}$ . The introduced orthogonality and projectivity constraints enhance spatial specificity, reducing overlap between components and facilitating interpretability (Sotiras et al., 2015, 2017).

However, challenges arise in high-dimensional scenarios, such as in MRI with millions of features. The covariance product $\text{X}{\text{X}}^{T}$ appearing in the numerator and denominator in Eq. (2) becomes too large to reside in memory. To address this, opNMF implementations have employed a memory-efficient update rule, which changes the order of multiplication and performs the calculation $\text{X}\left({\text{X}}^{T}\text{W}\right)$ in each iteration. Nevertheless, this strategy encounters further complications in large sample size studies, where the computational expense of computing the entire product $\text{X}{\text{X}}^{T}\text{W}$ in Eq. (2) becomes impractical. Consequently, these challenges render opNMF impractical for handling large neuroimaging datasets.

#### 2.1.2 Compressed stochastic opNMF (csopNMF)

Next, we will address the computational bottlenecks of opNMF by presenting our solution. We will begin by discussing the stochastic learning scheme, and then we will build upon this framework by incorporating mini-batch compression through structured random projections.

#### 2.1.3 Stochastic opNMF (sopNMF)

sopNMF is a variant of opNMF that aims to stochastically approximate the solution of opNMF over mini-batches of the data. By adopting the stochastic approach, we mitigate the impact of the costly term $\text{X}{\text{X}}^{T}\text{W}$ that appears twice in Eq. (2) by replacing it with ${\text{X}}_{p}{\text{X}}_{p}^{T}\text{W}$ , where ${\text{X}}_{p}\;\in {\mathbb{R}}_{+}^{m\times p}$ (p≪n ) is a small matrix constructed by randomly sampling subsets of the columns of the data matrix X (Z. Yang et al., 2012). The multiplicative update (MU) rule in Eq. (2) can be rewritten as

$${\text{W}}_{t+1}\leftarrow {\text{W}}_{t}\frac{{\left({\text{X}}_{p}{\text{X}}_{p}^{T}\text{W}\right)}_{t}}{{\left(\text{W}{\text{W}}^{T}{\text{X}}_{p}{\text{X}}_{p}^{T}\text{W}\right)}_{t}}.$$ (3)

The update rule in Eq. (3) can be thought of as the solution to the sequence of surrogate loss functions that minimize the opNMF objective over the mini-batches.

$$f_{n}\left(\text{W}\right)≜\frac{1}{B}\sum_{i=1}^{B}\ell \left({\text{X}}_{i},\text{W}\right),$$ (4)

where $\ell \;\left({\text{X}}_{i},\text{W}\right)$ is a loss function. For example, in the case of a single batch, we can write $\ell \;\left({\text{X}}_{p},\text{W}\right)$ as $l(X_{p},W)≜\underset{W\geq 0}{\min}X_{p}-WW^{T}X_{pF}^{\;2}.$.

In machine learning applications, we usually seek to minimize the expected cost $f\left(\text{W}\right)≜E_{x}\left[\ell \left(\text{X},\text{W}\right)\right]=$ $\lim_{n\to \infty }f_{n}\left(\text{W}\right)\;\text{a.s.}$ , where the expectation E is taken relative to the probability distribution of the data p(x) (Bottou, 2010; Mairal et al., 2010), instead of minimizing the empirical cost $f_{n}\left(\text{W}\right)$ with high accuracy. Considering a dataset composed of subjects from a distribution p(x), we draw a mini-batch ${\text{X}}_{i}$ and update ${\text{W}}_{t+1}$ by minimizing the function

$${\hat{f}}_{B}\left(\text{W}\right)≜\frac{1}{B}\sum_{i=1}^{B}\left({\left\|{\text{X}}_{i}-\text{W}{\text{W}}^{T}{\text{X}}_{i}\right\|}_{F}^{2}\right).$$

*Proposition 1* (Bani et al., 2023): *The sequence of surrogate objective functions converges almost surely*. ${\hat{f}}_{t}$ *acts as a converging surrogate of* f, *and* $f\left({\text{W}}_{t}\right)-{\hat{f}}_{B}\left({\text{W}}_{t}\right)\overset{\text{a.s.}}{\to }0$ *and hence* $f\left({\text{W}}_{t}\right)\overset{\text{a.s.}}{\to }0$ . This means that $\hat{f}$ is a surrogate and upper-bounds the empirical cost. Given that $\hat{f}$ acts as a surrogate of f, we can iterate over mini-batches of the data.

#### 2.1.4 Diversified sopNMF (dsopNMF)

Random sampling may lead to batches that contain redundant data that do not represent the population well, which may lead to biased results (Kulesza & Taskar, 2012; Zhang et al., 2017). DPPs are a repulsive sampling technique that aim to obtain samples that are as diverse as possible by encoding negative correlations (Kulesza & Taskar, 2012). The dissimilarity between the samples is measured using a real, symmetric, positive semi-definite (PSD) kernel $\text{L}\in {\mathbb{R}}^{n\times n}$ , where each element ${\text{L}}_{ij}$ is a measure of dissimilarity between samples i and j. In a DPP, subsampling any subset X of {1, …, n} is proportional to the determinant of the submatrix that indexes the subset ${\text{L}}_{X}$ of L:

$$P\left(X\right)=\frac{\det\left({\text{L}}_{X}\right)}{\det\left(\text{L}+\text{I}\right)}\propto \text{det}\left({\text{L}}_{X}\right).$$

We use κ-DPPs (Kulesza & Taskar, 2011) and condition the inclusion probability on the size of the subset $P_{\;\text{L}}^{\text{k}}\;\left(X\right)=\frac{\det\left({\text{L}}_{X}\right)}{\sum_{\left|X^{\prime }\right|}\text{det}\left({\text{L}}_{X^{\prime }}\right)}$, where |X|=p (in keeping with convention, and at the risk of abuse of notation, we use “κ” to refer to the number of subsets generated by the κ-DPPs sampling scheme). In our experiments, we create a linear kernel designed to introduce diversity among the batches, considering the similarities among the images of subjects in the study ${\text{L}}_{ij}={\text{x}}_{i}^{T}{\text{x}}_{j}$. sopNMF and dsopNMF are shown in Algorithm 1.

**Table:** 

**Algorithm 1:** sopNMF and dsopNMF
**Data:** data matrix $\text{X}\in {\mathbb{R}}_{+}^{m\times n}$ , target rank $k\in {\mathbb{N}}^{+}$, batch size p<<n , the number of batches $B=\frac{n}{p}$, initial guess of $\text{W}\in {\mathbb{R}}_{+}^{m\times r}={\text{W}}_{o}$, stopping criterion, max epoch, **Bool:** DPP **Result:** factorization matrix $\text{W}\in {\mathbb{R}}_{+}^{m\times r}$ **while** *stopping == False or epoch* < *max epoch* **do** **for** t=1 **to** *B* **do** **if** DPP *== True* **then** Read DPP mini-batch ${\text{X}}_{p}\;\in {\mathbb{R}}_{+}^{m\times p}$ ; **else** Read random mini-batch ${\text{X}}_{p}\;\in {\mathbb{R}}_{+}^{m\times p}$ ; **end** Update ${\text{W}}_{t+1}$ using Eq. (3); Record batch loss ${\left\|{\text{X}}_{p}-{\text{W}}_{t+1}{\text{W}}_{t+1}^{T}{\text{X}}_{p}\right\|}_{F}^{2}$; **end** calculate loss $=\frac{1}{B}\sum_{i=1}^{B}{\left\|{\text{X}}_{i}-{\text{W}}_{t+1}{\text{W}}_{t+1}^{T}{\text{X}}_{i}\right\|}_{F}^{2}$; **if** *Stopping == True* **then** Stop; **else** ${\text{W}}_{t}={\text{W}}_{t+1}$ ; **end** **end**

Depending on the analyzed data, the mini-batch diversification approach could be beneficial in two major ways. From a population-level perspective, DPPs allow us to capture mini-batches that are representative of the populations. From an optimization point of view, DPPs can lead to faster decay in the error as they reduce the variance in the stochastic updates.

*Proposition 2* (Bani et al., 2023): *The stochastic opNMF* κ-DPP *cost function* $f^{d}\left(\text{W}\right)$ *is a re-weighted empirical cost with* κ-DPP *weights* $\pi_{i}$

$$f^{d}\left(\text{W}\right)=\frac{1}{\kappa }\sum_{i=1}^{\kappa }\pi_{i}\ell \left({\text{X}}_{i},\text{W}\right).$$

*Proposition 3* (Bani et al., 2023): $f^{d}$ *results in variance reduction in the empirical updates*. *The product of the gradients of* ${\text{X}}_{i}$ *and* ${\text{X}}_{j}$ *is always positive whenever the correlation* $\sum_{ij}$ *is negative*

$$\forall_{i\neq j}:\sum_{ij}{\left[\frac{\partial \ell \left({\text{X}}_{i},\text{W}\right)}{\partial \text{W}}\right]}^{T}\left[\frac{\partial \ell \left({\text{X}}_{j},\text{W}\right)}{\partial \text{W}}\right]<0.$$

#### 2.1.5 Compressed sopNMF (csopNMF)

Stochastic optimization for opNMF provides a framework that allows the application of opNMF in large-scale settings and overcomes the memory constraints (Bani et al., 2023). However, stochastic optimization converges in sub-linear time (Bani et al., 2023; Mairal et al., 2010), and performing opNMF updates on mini-batches of hundreds of subjects (instead of the full study with thousands of subjects) is reminiscent of applying opNMF in moderate scale studies where it has been shown that the convergence is still very slow and can take up to weeks of compute time (Sotiras et al., 2015). The long compute times can be an insurmountable problem for research teams with limited resources as running time can be very expensive. To further improve the runtime and convergence speed of sopNMF, we accelerate the update steps using structured random projections (Halko et al., 2011; Tepper & Sapiro, 2016). We derive a compression algorithm (Algorithm 2) (Halko et al., 2011; Tepper & Sapiro, 2016) tailored for opNMF and integrate it into the stochastic learning scheme. Using random projections, we identify a low-dimensional subspace that retains most of the variance of each high-dimensional mini-batch and then project the mini-batch onto this subspace. Random projections enable computation in the compressed space while approximately preserving distances in the original space (Halko et al., 2011; Johnson et al., 1986).

**Table:** 

**Algorithm 2:** Compression algorithm for opNMF
**Data:** mini-batch (or full data matrix) $\text{X}\in {\mathbb{R}}^{m\times p}$ , target rank $k\in {\mathbb{N}}^{+}$, oversampling parameter $k_{ov}\;\in {\mathbb{N}}^{+}$, power iterations z **Result:** Compression matrix $\text{R}\in {\mathbb{R}}^{\left(k+k_{ov}\right)\times p}$ Draw ${\text{Ω}}_{R}\;\in {\mathbb{R}}^{\left(k+k_{ov}\right)\times m}$ ; Obtain the product $\xi ={\text{Ω}}_{R}{\text{X}}_{p}{\left({\text{X}}_{p}^{\text{T}}{\text{X}}_{p}\right)}^{z}$; Perform QR decomposition to obtain orthogonal basis for ξ

Accordingly, we present a new set of update rules that allows us to perform opNMF on compressed mini-batches while achieving close to optimal error in the uncompressed solution. We begin by forming Gaussian random matrix ${\text{Ω}}_{R}\;\in {\mathbb{R}}^{\left(k+k_{ov}\right)\times m}$ , where k is the opNMF target rank and $k_{ov}$ is an oversampling parameter that grants higher freedom for the compression (Halko et al., 2011). Using a uniformly sampled subset ${\text{X}}_{p}$ of the data matrix, we can obtain compression matrix $\text{R}\in {\mathbb{R}}^{\left(k+k_{\text{ov}}\right)\times p}$ using QR decomposition of the product ${\text{Ω}}_{R}{\text{X}}_{p}{\left({\text{X}}_{p}^{\text{T}}{\text{X}}_{p}\right)}^{z}$, where z is an exponent that performs power iterations that help with error reduction (Tepper & Sapiro, 2016). The compression matrix R is used to obtain a compression on a stochastic sample:

$${\tilde{\text{X}}}_{p}\;={\text{X}}_{p}{\text{R}}^{\text{T}}.$$ (5)

As demonstrated below, compressing with compression matrix R yields an error that tends toward the minimum achievable error (Halko et al., 2011; Tepper & Sapiro, 2016).

*Theorem 1* ((Halko et al., 2011)): *Consider a data matrix* $\text{X}\in {\mathbb{R}}^{m\times n}$ *, and target rank* k*, an oversampling parameter* $k_{ov}$ *where* $k+k_{ov}\;\leq \text{min}\left(p,m\right)$*, and* E *the expectation w.r.t. random matrix* Ω*. We obtain* R∈ℝ *by performing Algorithm 2 without power iterations (z=0 )*

$$E\left\|{\text{X}}_{p}-{\text{X}}_{p}{\text{R}}^{T}\text{R}\right\|\leq \left[1+\frac{4\sqrt{k+k_{ov}}}{k_{ov}-1}\cdot \sqrt{\text{min}\left\{m,p\right\}}\right]\sigma_{k+1},$$ (6)

*where* $\sigma_{k+1}$ *is the* ${\left(k+1\right)}^{th}$ *singular value of* ${\text{X}}_{p}$ *and is the smallest possible error achievable with any basis matrix* R.

Theorem 1 asserts that the compression algorithm yields an error that, on average, is within a small polynomial factor of the minimum achievable error, diminishing with $k_{ov}$ . Additionally, owing to concentration of measure effects, the realized error is almost always in proximity to the theoretical minimum (Halko et al., 2011). The likelihood that the error meets the condition $\text{X}p-\text{X}p{\text{R}}^{T}\text{R}\leq \left[1+9\sqrt{k+k_{ov}}\;\cdot \;\sqrt{\text{min}\left\{m,p\right\}}\right]\sigma_{k+1}$ is at least $1-3k_{ov}^{-k_{ov}}$ , justifying the use of a relatively small $k_{ov}$ , such as 5 (Halko et al., 2011; Martinsson & Voronin, 2016).

We can now obtain a compressed stochastic version of Eq. (1), where at each batch we solve the reduced sub-problem:

$$\underset{W\geq 0}{\text{min}}{\left\|{\tilde{\text{X}}}_{p}-\text{W}{\text{W}}^{T}{\tilde{\text{X}}}_{p}\right\|}_{F}^{2}\text{ s.t. }{\text{W}}^{T}\text{W}=I,$$ (7)

with update rule

$${\text{W}}_{t+1}\leftarrow {\text{W}}_{t}\frac{{\left({\tilde{\text{X}}}_{p}{\tilde{\text{X}}}_{p}^{T}\text{W}\right)}_{t}}{{\left(\text{W}{\text{W}}^{T}{\tilde{\text{X}}}_{p}{\tilde{\text{X}}}_{p}^{T}\text{W}\right)}_{t}}.$$ (8)

This approach, termed compressed stochastic opNMF or csopNMF (Algorithm 3), yields significant speed up in the calculations by effectively reducing the columns of each batch from p to ($k+k_{ov}$ ). It is also important to note that opNMF is not dependent on a learning rate because it emulates a rescaled gradient descent. However, this approach grants us the freedom to pass over the data multiple times creating epochs that can tune the learning of the algorithm.

**Table:** 

**Algorithm 3:** csopNMF
**Data:** data matrix $\text{X}\in {\mathbb{R}}_{+}^{m\times n}$ , target rank $k\in {\mathbb{N}}^{+}$, oversampling parameter $k_{ov}\;\in {\mathbb{N}}^{+}$, batch size p<<n , the number of batches $B=\frac{n}{p}$, initial guess of $\text{W}\in {\mathbb{R}}_{+}^{m\times k}\;={\text{W}}_{o}$, stopping criterion, max epoch, number of inner iterations iter , **Bool:** DPP **Result:** factorization matrix $\text{W}\;\in {\mathbb{R}}_{+}^{m\times k}$ **while** *stopping == False or epoch* < *max epoch* **do** **for** t=1 **to** *B* **do** **if** DPP *== True* **then** Read DPP mini-batch ${\text{X}}_{p}\;\in {\mathbb{R}}_{+}^{m\times p}$ ; compute compression matrix $\text{R}\in {\mathbb{R}}_{+}^{k+k_{ov}\times p}$ using Algorithm 2; compute ${\tilde{\text{X}}}_{p}\;\in {\mathbb{R}}_{+}^{m\times k+k_{ov}}\;={\text{X}}_{p}{\text{R}}^{T}$; **else** Read random mini-batch ${\text{X}}_{p}\;\in {\mathbb{R}}_{+}^{m\times p}$ ; compute compression matrix $\text{R}\in {\mathbb{R}}_{+}^{k+k_{ov}\times p}$ using Algorithm 2; compute ${\tilde{\text{X}}}_{p}\;\in {\mathbb{R}}_{+}^{m\times k+k_{ov}}\;={\text{X}}_{p}{\text{R}}^{T}$; **end** **for** i=1 **to** *iter* **do** Update ${\text{W}}_{t+1}$ using Eq. (8); Record batch loss ${\left\|{\text{X}}_{p}\;-{\text{W}}_{t+1}{\text{W}}_{t+1}^{T}{\text{X}}_{p}\right\|}_{F}^{2}$; **end** **end** calculate loss $=\frac{1}{B}\sum_{i=1}^{B}{\left\|{\text{X}}_{i}\;-{\text{W}}_{t+1}{\text{W}}_{t+1}^{T}{\text{X}}_{i}\right\|}_{F}^{2}$; **if** *Stopping == True* **then** Stop; **else** ${\text{W}}_{t}\;={\text{W}}_{t+1;}$ **end** **end**

#### 2.1.6 Advantages for big neuroimaging data analyses

In contrast to previous compression frameworks, our approach focuses on compressing the mini-batches rather than directly compressing the entire input data matrix, although this is feasible in certain cases. This choice is motivated by two primary considerations. Firstly, in scenarios where the number of samples and features is substantial, such as in many large-scale neuroimaging studies, the matrix ξ (Algorithm 2) could become too large to store in memory, rendering QR decomposition impractical. Given our algorithm’s stochastic nature, it enables us to execute the necessary decomposition and compression without a significant loss of precision at the mini-batch level. Secondly, it avoids the complexity associated with distributed hardware optimization architectures such as MapReduce-based TSQR (Benson et al., 2013; Liu et al., 2010; Tepper & Sapiro, 2016).

### 2.2 Datasets

In this study we used two datasets. Open Access Series of Imaging Studies (OASIS) served as the validation cohort to benchmark our proposed csopNMF against opnmf and sopNMF. OASIS is large enough to demonstrate the advantages of our method, yet small enough to run standard opNMF for direct, head-to-head comparison. UK Biobank then provided a population-scale application: its size and memory demands make conventional opNMF computationally impractical, allowing us to show that the proposed method scales to big data without sacrificing interpretability.

#### 2.2.1 Validation dataset: Open access series of imaging studies (OASIS)

Data for method validation were T1-weighted MR images obtained from 1,000 subjects (mean age: 69.78±9.53 years, 550 female) sourced from the Open Access Series of Imaging Studies (specifically OASIS-3 release) dataset (LaMontagne et al., 2019). We selected OASIS because it is one of the largest publicly available neuroimaging datasets. Moreover, OASIS is sufficiently large to provide robust analysis while still being manageable for performing opNMF. Preprocessing involved gray matter (GM) segmentations via FSL FAST (Jenkinson et al., 2012), registering them to MNI152 template space via DRAMMS (Ou et al., 2011), modulating with the Jacobian determinant, and smoothing with a 6 mm full-width half-maximum Gaussian kernel. Subsequently, the smoothed GM tissue density maps were vectorized and arranged column wise to form the input data matrix $\text{X}\in {\mathbb{R}}_{+}^{1187150\times 1000}$ .

#### 2.2.2 Application dataset: UK Biobank (UKB)

Data used for method application consisted of T1-weighted MR images obtained from 10,000 healthy subjects (mean age: 63.82±7.68 years, 5243 female) sourced from UKB. The UKB sample included only participants without any International Classification of Diseases (ICD-10) diagnostic codes at baseline (UKB data field 41,270). This restriction ensured a healthy cohort and minimized potential confounding effects of pathology on brain structural organization. Preprocessing involved the following steps: (1) skull stripping with DeepMRSeg (Doshi et al., 2019); (2) gray matter segmentations using FSL FAST to obtain GM Partial-volume maps; (3) rigid, affine, and nonlinear registration of the T1w images to MNI152 1 mm template with ANTS, estimating the affine and warp fields; (4) application of the transforms to the GM maps to bring all data into template space; (5) computation of the deformation-field Jacobian determinant and Jacobian modulation of the GM maps to account for local expansion and contraction; (6) smoothing with 6 mm full-width at half maximum Gaussian Kernel. The smoothed GM tissue density maps were vectorized and arranged column wise to form the input data matrix $\text{X}\in {\mathbb{R}}_{+}^{1187150\times 10000}$ .

### 2.3 Experimental setup

This section details the materials and methods for both model validation and application. In the validation part, we describe the experimental setup, algorithm parameters, and comparison metrics used to benchmark csopNMF against opNMF and sopNMF. In the application part, we specify the configuration used to derive patterns of structural covariance (PSCs) and outline the downstream statistical analyses used to assess associations with phenotypes. We refer to the spatial components in W as PSCs, following prior neuroimaging literature; thus, PSCs and components refer to the same factorization outputs.

#### 2.3.1 Model validation on OASIS

We tested three configurations of csopNMF, each differing in the way the compression matrix is computed and the number of iterations performed for each mini-batch. We compared the performance of the csopNMF variants against both traditional opNMF and sopNMF. In the first csopNMF setup, we derived a compression matrix from each mini-batch, executed 10 iterations on the mini-batch, and repeated this process for the remaining batches. In the second csopNMF configuration, we generated a compression matrix from the entire data matrix and utilized it to compress various mini-batches. Subsequently, we performed a single iteration on each mini-batch. The third csopNMF configuration mirrors the second, with the distinction that we performed 10 iterations on each mini-batch in this case. For each of the 3 csopNMF, we tested both random sampling and DPP sampling with mini-batch sizes of 125 and 250. We also tested different oversampling parameters $k_{ov}\;=\left\{10,\;20,\;60\right\}$. Since we did not impose constraints on how often a subject was sampled, we ensured that all subjects were revisited at the end of the learning process to guarantee that the model had seen all of the data. For sopNMF, we matched the sampling to that of csopNMF and employed random sampling and DPP sampling with mini-batch sizes of 125 and 250. A summary of the different model parameters is shown in Table 1. In total, csopNMF had 36 different configurations and sopNMF had 4 different configurations.

**Table 1.** Summary of algorithm parameters.

Parameter	Value
NMF rank (k)	20
Sampling	Random, DPP
Batch size (p)	125, 250
Oversampling ($k_{ov}$ )	10, 20, 60
Compression scenarios	Mini-batch, full data
csopNMF iterations/batch	1, 10
Total number of iterations	200,000

For all of the experiments, we opted for a factorization rank k=20 commonly utilized in neuroimaging analyses to offer a low-dimensional representation of the brain (Damoiseaux et al., 2006; Pizzo et al., 2019; Smith et al., 2009; Sotiras et al., 2015, 2017; Yeo et al., 2014). For all methods, non-negative double singular value decomposition (NNDSVD) was selected for initialization due to its deterministic nature and faster convergence (Boutsidis & Gallopoulos, 2008). opNMF was executed until completion with 200,000 iterations. For csopNMF and sopNMF variants, iterations were divided into epochs based on the batch size, that is, $\frac{200,000}{n/p}$ , with a stopping criterion based on consecutive decreases in the loss function over the last 100 epochs. For our purposes, the stopping criterion was turned off to allow for a fair computational comparison against opNMF. All experiments were conducted with access to eight threads on Xeon Gold 6226R CPUs and 32 GB of DDR4 RAM. Implementations were performed in Python, and the codes will be made available at https://github.com/sotiraslab/csopNMF

#### 2.3.2 Model validation metrics

We validated our method on OASIS, where we baselined csopNMF against opNMF and its stochastic variant (sopNMF) in terms of computational burden (memory usage and compute time) reconstruction accuracy (reconstruction error), sparsity, and component reproducibility.

##### 2.3.2.1 Memory usage and compute time

We evaluated scalability by comparing the peak memory consumption of csopNMF and sopNMF during multiplicative updates against that of standard opNMF. In addition, we recorded the total wall-clock time required for each algorithm to run to completion.

##### 2.3.2.2 Reconstruction error

We quantified reconstruction fidelity using the Frobenius norm of the residual matrix

$${\left\|\text{X}-\text{WH}\right\|}_{F}^{2},$$

where X denotes the input data and WH is the low-rank approximation obtained from the factorization. Lower values of this error indicate a closer match between the approximation and the observed data, and thus better preservation of the underlying variance structure. We performed a comparative analysis of reconstruction error across opNMF, sopNMF, and csopNMF to assess whether the computational gains of the proposed methods were achieved without loss of approximation quality.

##### 2.3.2.3 Sparsity

Anatomical networks are expected to exhibit unique patterns of variability, and opNMF is able to decompose the input signal into distinct and non-overlapping parts (Sotiras et al., 2015). This property is of particular interest in neuroimaging and is reflected through component sparsity. For the component matrix $\text{W}=\left[{\text{w}}_{1},{\text{w}}_{2},\ldots ,{\text{w}}_{k}\right]$, with ${\text{w}}_{i}\in {\mathbb{R}}_{+}^{m}$, sparsity was defined as (Hoyer, 2004)

$$s\left(\text{w}\right)=\frac{\sqrt{m}-\left(\sum_{i=1}^{m}\left|{\text{w}}_{i}\right|\right)/\sqrt{\sum_{i=1}^{m}{\text{w}}_{i}^{2}}}{\sqrt{m}-1}\in \left[0,1\right],$$

where ${\text{w}}_{i}$ is the $i^{\text{th}}$ element of the vector w. Values of s closer to 1 indicate greater sparsity, corresponding to components with more localized and non-overlapping anatomical support. We computed sparsity for each component of W across opNMF, sopNMF, and csopNMF to evaluate whether computational gains in the proposed methods were achieved without loss of anatomical interpretability.

##### 2.3.2.4 Component reproducibility metrics

Because the spatial components constitute the primary object of interpretation in opNMF, it is essential that the proposed variants recover component structure consistent with the reference solution. Component reproducibility was quantified using the adjusted rand index (ARI) and the mean and median inner products between matched components. ARI measures agreement between two clustering assignments beyond chance, with values in [−1, 1] and higher values indicating stronger similarity. Inner products were computed between matched component vectors and take values in [0, 1], with higher values reflecting greater correspondence. A hard winner-takes-all (WTA) clustering was obtained by assigning each voxel to the component with the highest loading to elimante overlap and to eliminate overlap and derive a composite visualization that is more economic and compact. Similarity metrics were computed between each variant and the opNMF solution.

##### 2.3.2.5 Loading coefficient reproducibility assessed via brain-age prediction

To assess whether the loading coefficients produced by the proposed variants preserve downstream utility of opNMF, we evaluated their performance in a standard brain-age regression task. In the context of NMF, the component matrix W provides a latent spatial basis, while the loading matrix H encodes how each subject is expressed in that latent space. These coefficients are widely used as features in brain-age prediction because they capture interpretable, low-dimensional structure relevant to normative aging trajectories (Varikuti et al., 2018).

We trained support vector regression (SVR) models to predict chronological age from loading coefficients derived from opNMF, csopNMF, and sopNMF. For the stochastic variants, we evaluated reproducibility using the configurations that achieved the highest and lowest ARI values, ensuring that downstream performance was examined under both best- and worst-alignment conditions. Model performance was estimated using 10 realizations of 10-fold cross-validation. For each fold and realization, we recorded the coefficient of determination ($R^{2}$) and mean absolute error (MAE). Statistical comparison between methods was performed using a one-way ANOVA on the cross-validated $R^{2}$ and MAE distribution. Finally, as an exploratory cognitive validation analysis, we additionally examined whether the brain-age gap derived from the opNMF and csopNMF loading coefficients was associated with MMSE scores using Spearman correlation.

#### 2.3.3 Applying csopNMF to UKB

To demonstrate the utility of csopNMF in large-scale neuroimaging analysis, we applied it to a subset of 10,000 UKB subjects to derive a set of 20 data-driven components, referred to as patterns of structural covariance (PSCs). We characterized these based on their approximate anatomical location within the brain. We then tested associations between PSC loadings and visceral adipose tissue to investigate links between metabolic health and brain structure. Finally, we showed how these patterns refine in higher-rank decompositions with 40 and 60 components.

##### 2.3.3.1 PSCs from the UKB

We estimated k=20 patterns of structural covariance (PSCs) (Damoiseaux et al., 2006; Pizzo et al., 2019; Smith et al., 2009; Sotiras et al., 2015, 2017; Yeo et al., 2014) from 10,000 UK Biobank participants using csopNMF with random sampling, a mini-batch size of p=125 , and oversampling $k_{\text{ov}}\;=20$ . PSCs were visualized using Surfice (Rorden, 2025) and FSL (Jenkinson et al., 2012), and each pattern was described based on its approximate anatomical coverage. To assess the stability of the decomposition under stochastic sampling, we repeated the analysis five times using independent random seeds and quantified pairwise agreement between solutions using the ARI and the mean and median component inner products.

##### 2.3.3.2 Statistical analysis

Our goal was to test whether VAT relates to brain morphology summarized by data-driven csopNMF PSCs. To this end, we fitted linear regressions to predict PSC loadings from VAT, age, and their interaction. To isolate sex-specific effects, separate models were fitted for males and females. To minimize confounding by head size, PSC loadings were first normalized by intracranial volume (ICV). UK Biobank phenotypes (VAT, age, sex, ICV) were aligned to imaging by participant ID, and only subjects with complete data were retained. Subjects without VAT information were removed. VAT outliers were removed within sex using a Tukey interquartile range fence. Age and VAT were z-scored to place effects on a comparable scale and stabilize estimation. The final number of subjects included in the statistical analysis was 8260. Multiple comparisons across PSCs were addressed with false discovery rate (FDR) correction within each sex.

##### 2.3.3.3 Finer PSCs from the UKB and higher order models

Our main analyses used rank parameter k=20 to offer a low-dimensional representation of the brain, following previous neuroimaging analyses (Damoiseaux et al., 2006; Pizzo et al., 2019; Smith et al., 2009; Sotiras et al., 2015, 2017; Yeo et al., 2014). We additionally examined rank 40 and rank 60 solutions (with mini-batch sizes of p=250 and oversampling $k_{\text{ov}}\;=60$ and 80 , respectively) to further illustrate the behavior of the decomposition across model orders.

## 3 Results

### 3.1 Model validation on OASIS: csopNMF vs. opNMF and sopNMF

Our hypothesis is that csopNMF can emulate the performance of opNMF in generating comparable structural covariance patterns while requiring fewer computational resources. To verify this hypothesis, we evaluated various configurations of csopNMF against opNMF in terms of reconstruction error, sparsity, computation time, memory usage, and resultant patterns. We also included the stochastic-only version, sopNMF, in the comparison. The similarity between the resulting patterns was quantified using the adjusted rand index, mean and median inner products, and further examined visually. Finally, we demonstrated that the factorizations produced by csopNMF and opNMF are equally effective for downstream analyses by using their coefficients in a standard machine learning task of brain-age regression.

#### 3.1.1 Memory usage and compute time

**Fig. 3.**
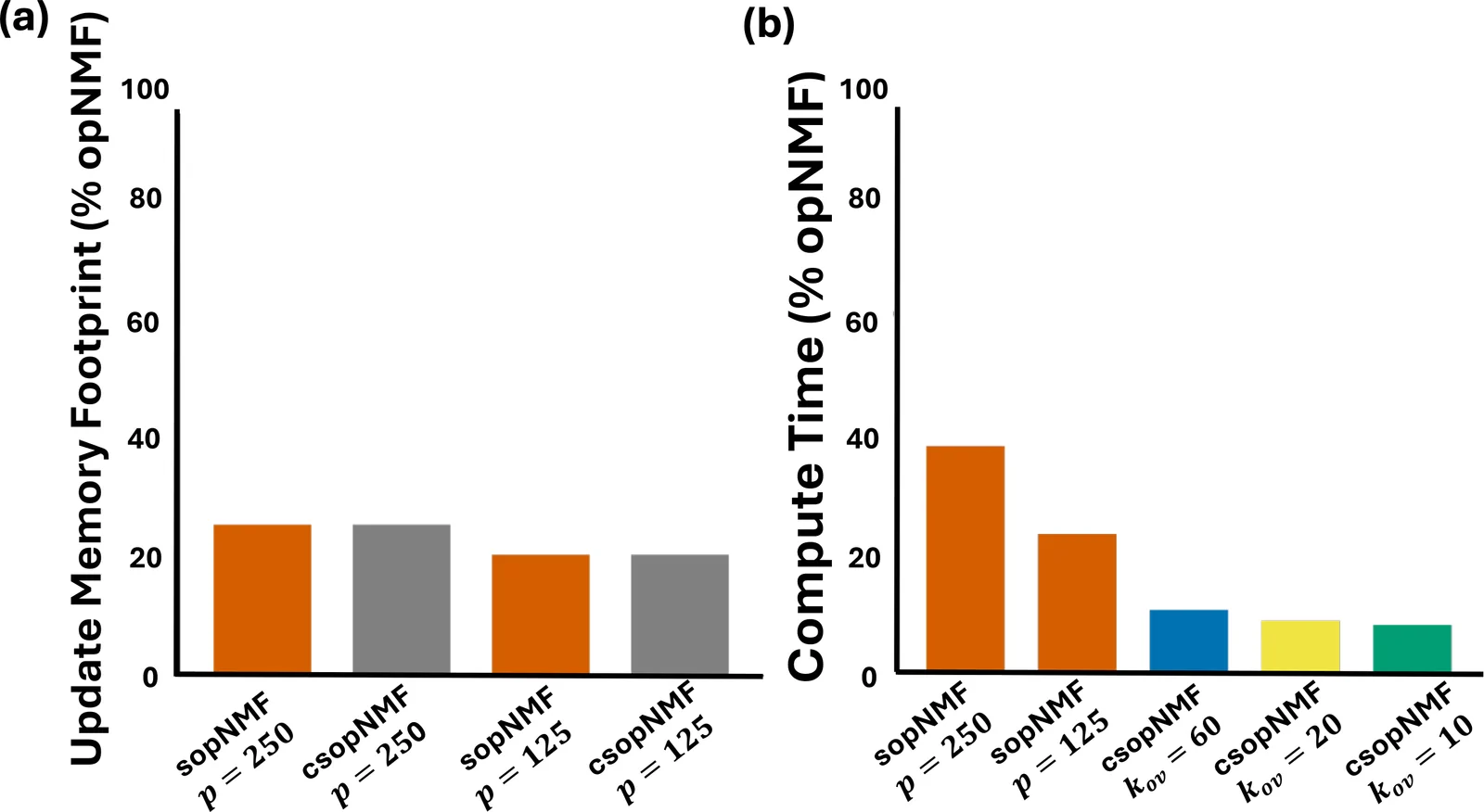
Relative memory usage (a) and compute time (b) of sopNMF and csopNMF compared with opNMF are reported. At different mini-batch sizes, both csopNMF and sopNMF use 25–25% of the memory required by the memory-optimized version of opNMF. For the same memory footprint, csopNMF reduces the compute time of opNMF by over 80%.

We evaluated the scalability of csopNMF and sopNMF by comparing their peak memory usage during the multiplicative updates with that of opNMF. For the largest batch size, p=250 , csopNMF and sopNMF used less than 25% of the memory required by opNMF, with even lower memory usage observed for the smaller batch size of p=125 (Fig. 3a). This reduction in memory consumption also came with increased efficiency. To assess this, we compared the total runtime of csopNMF and sopNMF with opNMF over 200,000 iterations of the multiplicative update. csopNMF was approximately 90% faster than opNMF at the lowest $k_{ov}$ and 85% faster at the highest $k_{ov}$ (Fig. 3b). Similarly, sopNMF was 60% faster for the larger batch size and around 70% faster for the smaller batch size (Fig. 3b). However, sopNMF required longer computation times with larger mini-batches, as it takes more time to complete the same number of iterations. It is important to note that sampling introduces some overhead. While this overhead is negligible for random sampling, DPP sampling can increase the algorithm’s runtime due to the eigendecomposition required for the DPP kernel. Nevertheless, efficient sampling methods are available, such as projecting the kernel onto a lower-dimensional space (Affandi et al., 2013) or minimizing the total variation distance between the original DPP and an approximation (C. Li et al., n.d.), which can substitute exact DPP sampling in our framework. Additionally, sampling can be performed independently of the algorithm and invoked when required. The decision to use DPP sampling should be informed by the specific research question and the characteristics of a study’s cohort to justify the additional computational burden.

#### 3.1.2 Reconstruction error

**Fig. 4.**
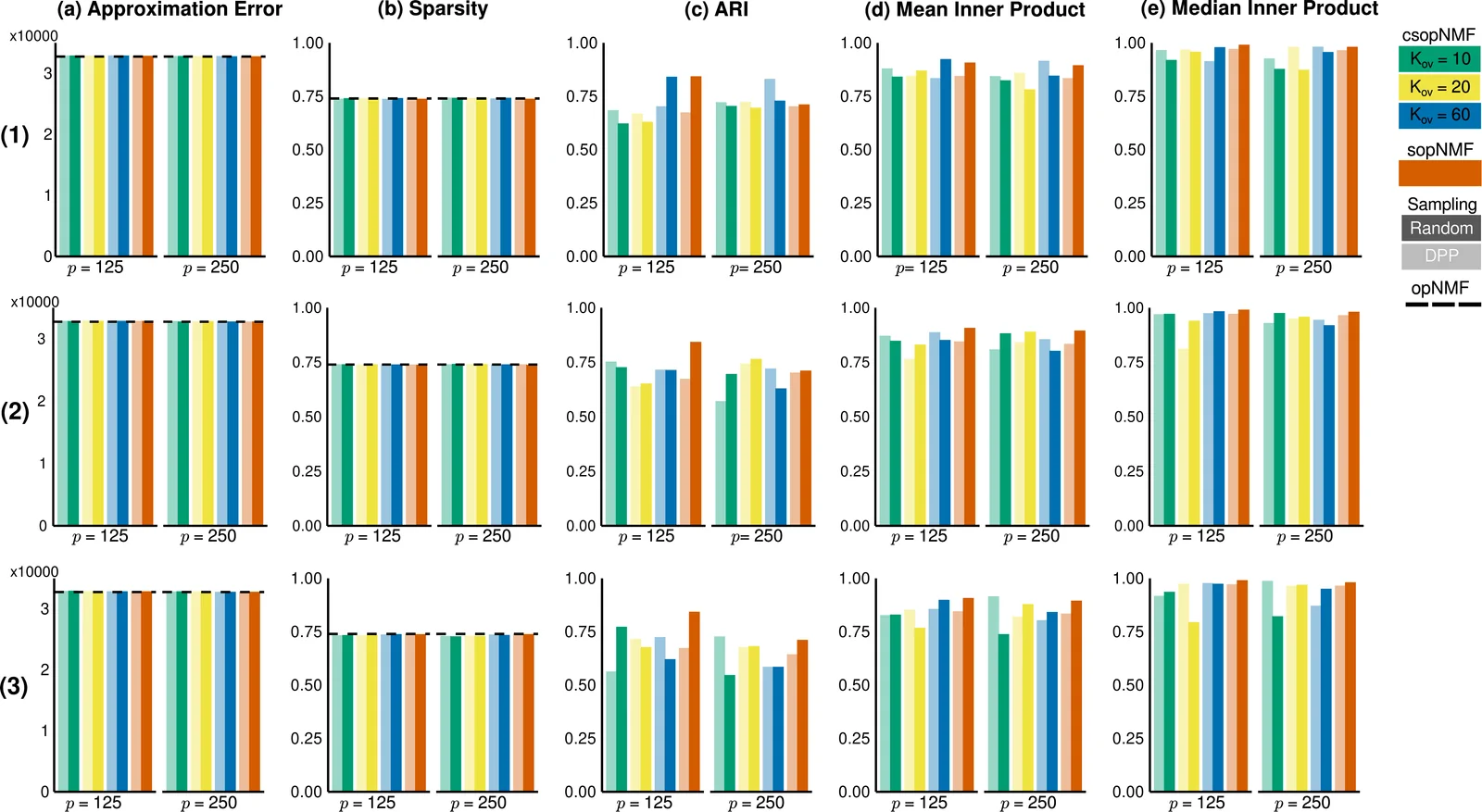
Performance comparison of the proposed variants compared with opNMF. Each row corresponds to a different setup. Row (1) corresponds to obtaining a compression matrix from the full data and performing a single iteration on the compressed mini-batch; row (2) corresponds to obtaining a compression matrix from the full data and performing 10 iterations on the compressed mini-batch; while row (3) corresponds to obtaining the compression matrix from each mini-batch and performing 10 iterations on the compressed mini-batch. The columns represent the following performance metrics evaluated at two different mini-batch sizes (p=125, p=250 ): (a) approximation error ( × 10,000), (b) sparsity, (c) adjusted rand index (ARI), (d) mean inner product, and (e) median inner product. In each subplot and from left to right, the first three bars represent different csopNMF compressions: $k_{ov}\;=10$ (green), $k_{ov}\;=20$ (yellow), $K_{ov}\;=60$ (blue), while the fourth corresponds to sopNMF (orange, same across all plots). Determinantal point processes (DPP) variants are shown with higher transparency for all variants. opNMF error and sparsity are shown as a dashed line. ARI, mean, and median inner products are obtained relative to opNMF.

Because the stochastic and compressed variants are designed to approximate the opNMF solution, we compared reconstruction error across methods. In all tested configurations, csopNMF and sopNMF achieved reconstruction errors comparable with those of opNMF, with no appreciable increase relative to the reference solution (Fig. 4a), despite the substantially lower computational cost of the proposed variants.

#### 3.1.3 Sparsity

Because interpretability in opNMF is reflected in the spatial sparsity of its components, we assessed whether this property was preserved in the proposed variants. Across all configurations, csopNMF and sopNMF achieved sparsity values exceeding 0.7, matching the spatial localization observed in opNMF (Fig. 4b), indicating that the stochastic and compressed formulations maintain the anatomical specificity characteristic of opNMF.

#### 3.1.4 Reproducibility

The goal of the stochastic optimization and compression process is to accurately approximate the solution of opNMF. However, we also aim with our solution to replicate the components and coefficients generated by opNMF.

##### 3.1.4.1 Component similarity

**Fig. 5.**
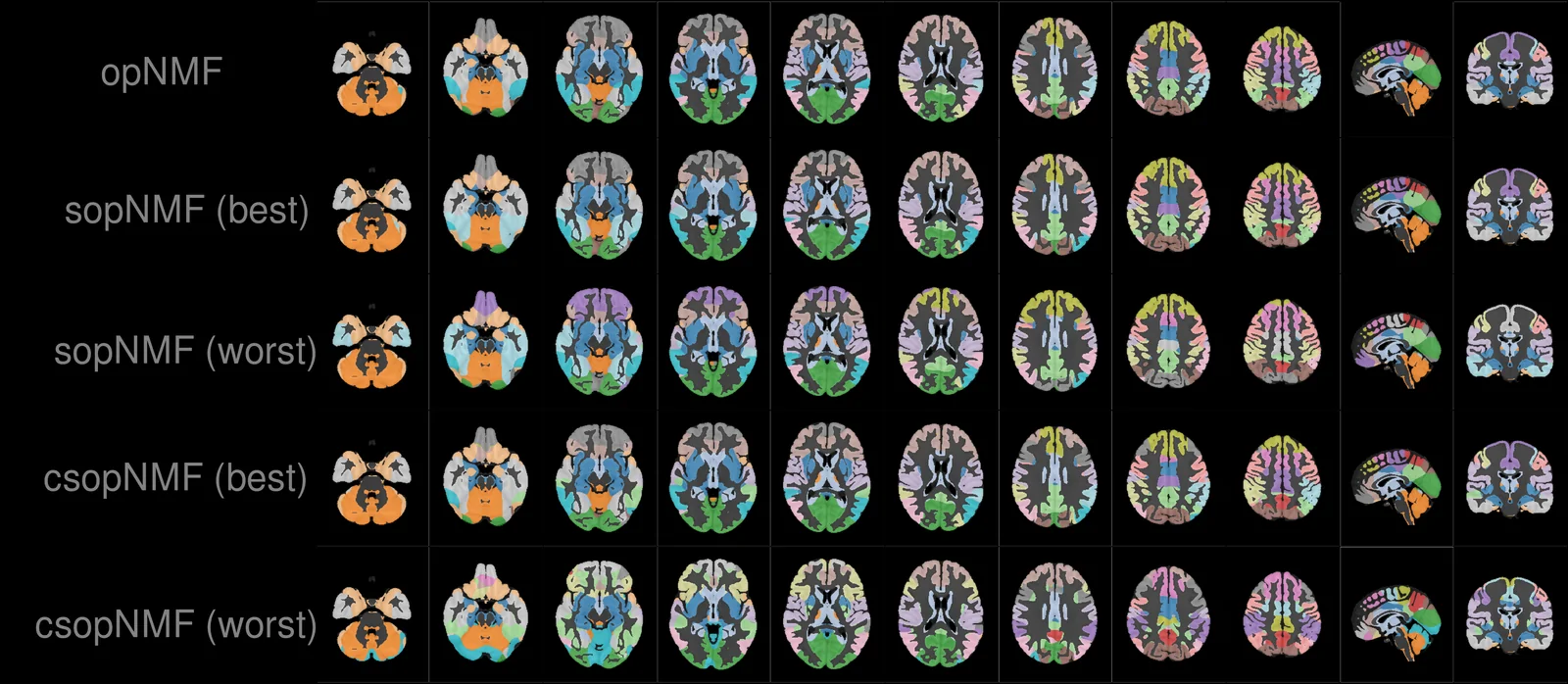
Visualization of typical opNMF components produced the winner-takes-all (WTA) approach offering a compact visualization of all the components. The first row shows the components generated by the baseline opNMF. The second and third rows show the components of sopNMF corresponding to the setup with the highest ARI of 0.84 (best) and the setup corresponding to the lowest ARI of 0.65 (worst). The fourth and fifth rows show the components of csopNMF corresponding to the setup with the highest ARI of 0.87 (best) and the setup corresponding to the lowest ARI of 0.50 (worst). For sopNMF, the highest ARI (0.84) was achieved with a mini-batch size of p=125 , sampled uniformly. The lowest ARI for sopNMF (0.654) was achieved with a mini-batch size of p=125 , DPP sampled. For csopNMF, the highest ARI (0.87) was achieved with a mini-batch size of p=125 , sampled uniformly, and compressed using a compression matrix derived from the full dataset, with an oversampling parameter of $k_{ov}\;=60$ , and a single iteration per batch. The lowest ARI for csopNMF (0.49) was achieved with a mini-batch size of p=250 , sampled uniformly, and compressed using a compression matrix derived from the mini-batch, with an oversampling parameter of $k_{ov}\;=10$ , and 10 iterations per batch.

Across all tested configurations, both csopNMF and sopNMF recovered components that closely matched those obtained by opNMF. ARI values consistently exceeded 0.5, indicating strong agreement in voxel-wise assignments (Fig. 4c). Likewise, the mean and median inner products between matched components were above 0.7 for all configurations (Fig. 4d–e), confirming high correspondence in spatial patterns. Visual inspection of representative components further corroborated the quantitative similarity (Fig. 5), with representative results shown for each method at the configurations achieving the highest and lowest ARI values.

##### 3.1.4.2 Loading coefficient reproducibility assessed via brain-age prediction

**Fig. 6.**
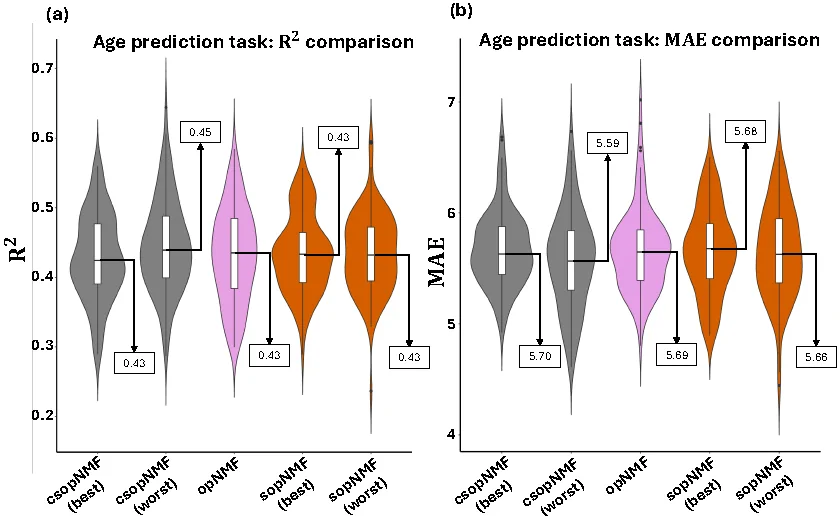
Performance in brain-age prediction: The ${\text{R}}^{2}$ and MAE values are reported for brain-age prediction task, using 10 realizations of 10-fold cross-validated OASIS-3 data. Coefficients H from the csopNMF and sopNMF configurations with the highest and lowest ARI for each method were used in support vector regression (SVR) to predict each subject’s brain age. The results were compared with those from opNMF. (a) The ${\text{R}}^{2}$ values for csopNMF and sopNMF are similar to those of opNMF, with no statistically significant differences. (b) Prediction errors (MAE) for csopNMF and sopNMF are also comparable with those of opNMF, again showing no statistically significant differences.

Across all models, loading coefficients derived from csopNMF and sopNMF yielded brain-age prediction performance that was statistically indistinguishable from that obtained using opNMF coefficients. The cross-validated mean absolute error (MAE) ranged from 5.59 to 5.70 years and the coefficient of determination ($R^{2}$) ranged from 0.43 to 0.45 across methods. One-way ANOVA revealed no significant differences in either $R^{2}$ or MAE across opNMF, csopNMF, and sopNMF (Fig. 6a–b), indicating that the stochastic and compressed variants preserve downstream predictive utility. Finally, in an exploratory cognitive validation analysis, age-bias-corrected brain-age gap showed significant negative association with MMSE for both csopNMF (r=−0.197 , p− value <0.001 ) and opNMF (r=−0.194 , p− value <0.001 ) (Supplementary Material S1 Fig. S1), indicating that higher brain-age gap was associated with lower cognitive performance.

### 3.2 Application of csopNMF to UK Biobank

In this study, we developed and validated csopNMF, a scalable and efficient extension of opNMF that uses stochastic learning and data compression. In the subsequent analysis, we applied csopNMF to GM density maps from 10,000 UKB participants to derive 20 PSCs representation of the brain and examine associations between structural integrity and visceral adipose tissue. To further illustrate the flexibility of the framework across different ranks, we also examined higher-rank csopNMF decompositions in the UKB data and extracted finer-grained networks of 40 and 60 PSCs, complementing our rank 20 solution.

#### 3.2.1 Characterizing structural parcellation

**Fig. 7.**
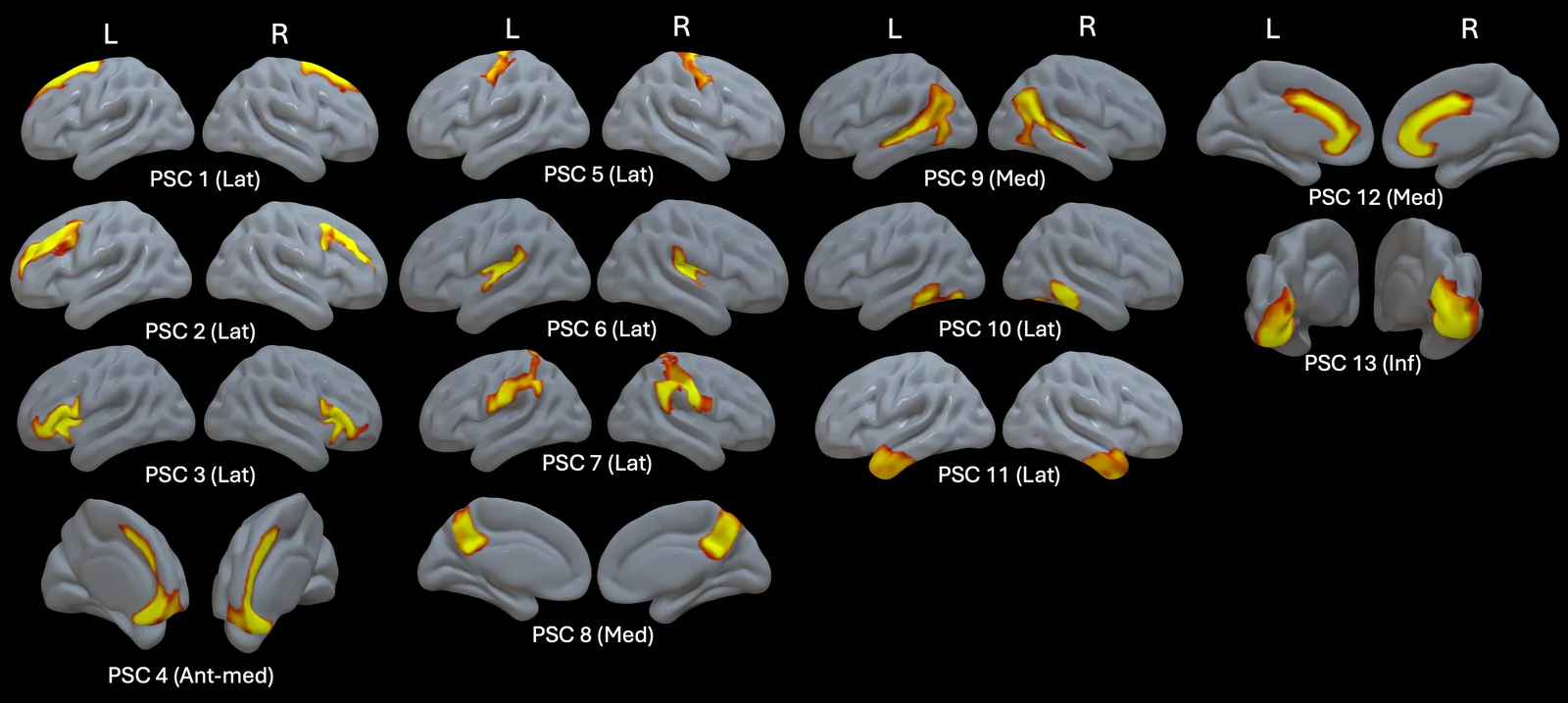
Cortical patterns of structural covariance (PSCs) estimated with csopNMF, arranged by lobe for readability. Frontal cortex is represented by PSC 1 (dorsal), PSC 2 (middle frontal), PSC 3 (ventrolateral), and PSC 4 (medial frontal and orbitofrontal). Parietal territories include PSCs 5–6 (perirolandic and posterior insular cortex), PSC 7 (postcentral), and the PSC 8 (precuneus). Temporoparietal junction and superior temporal cortex are represented by PSC 9, while the temporal lobe is summarized by PSC 10 (inferior temporal) and PSC 11 (temporal pole). PSC 12 highlights the cingulate gyrus and PSC 13 covers occipital cortex.

**Fig. 8.**
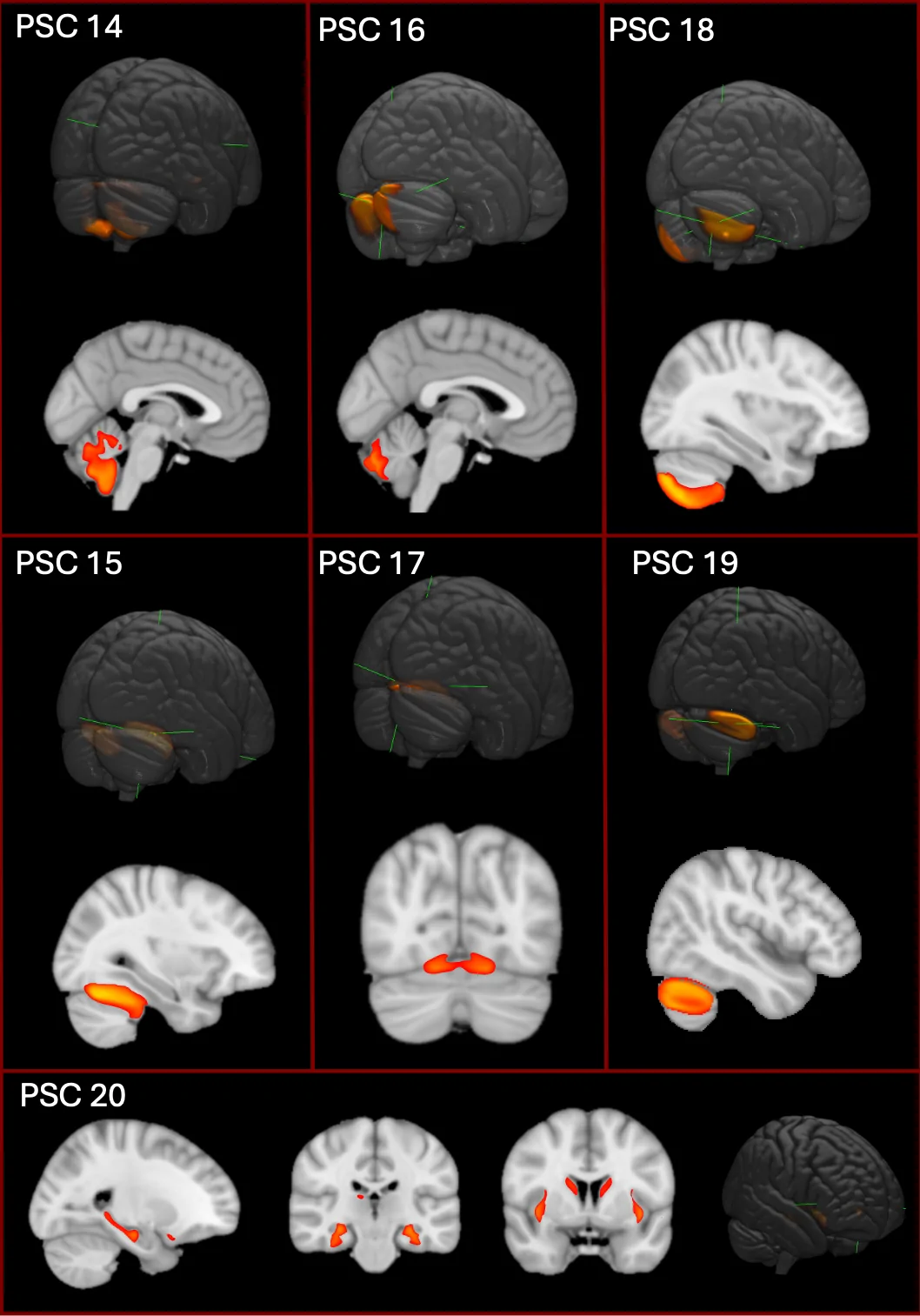
Spatial maps of cerebellar and subcortical patterns of structural covariance (PSCs) in MNI152 space. PSCs 14–19 delineate cerebellar territories (medial/intermediate zones and lateral hemispheres), and PSC 20 captures subcortical nuclei, including hippocampus and basal ganglia (striatum, thalamus, putamen, globus pallidus).

The non-negative parcellation from csopNMF provides an additive, parts-based representation of brain regions. The orthonormality and projective constraints, alongside non-negativity, encourage minimally overlapping, soft clustering of regions in a data-driven manner. A visual inspection of the components shows their complementary nature, with minimally overlapping patterns (Figs. 7 & 8). The parcellations can be grouped into three sets according to their approximate anatomical coverage: cortical, cerebellar, and subcortical. PSCs 1 to 13 summarize cortical patterns (Fig. 7), PSCs 14 to 19 decompose the cerebellum (Fig. 8), and PSC 20 captures subcortical structures (Fig. 8). Within the cortex, the frontal lobe is represented by PSC 1 (dorsal), PSC 2 (middle frontal), PSC 3 (ventrolateral), and PSC 4 (medial frontal and orbitofrontal) (Fig. 7). The parietal lobe is represented by PSCs 5–8, covering the precentral (PSC 5), supramarginal (PSC 6), postcentral (PSC 7) gyri, and the precuneus (PSC 8) (Fig. 7). The temporoparietal junction and superior temporal cortex are captured by PSC 9 (Fig. 7). The remaining temporal lobe is summarized by PSC 10 (inferior temporal) and PSC 11 (temporal pole) (Fig. 7). PSC 12 highlights the cingulate gyrus, and PSC 13 covers the occipital lobe (Fig. 7). The cerebellum is represented by 6 PSCs: PSCs 14, 16, and 17 cover medial and intermediate zones, while PSCs 18, 15, and 19 cover lateral hemispheric regions (Fig. 8). PSC 20 summarizes key subcortical nuclei, including the hippocampus and basal ganglia (striatum, thalamus, putamen, and globus pallidus) (Fig. 8). Stability analysis across five runs initialized with independent random seeds showed consistently high agreement among the resulting decompositions, as measured by the ARI (0.75±0.06 ), mean (0.91±0.05 ), and median (0.97±0.02 ) component inner products, demonstrating that the identified PSCs were robust to stochastic sampling (Supplementary Material S2 Fig. S2).

#### 3.2.2 Associations between PSC loadings and visceral adipose tissue

**Fig. 9.**
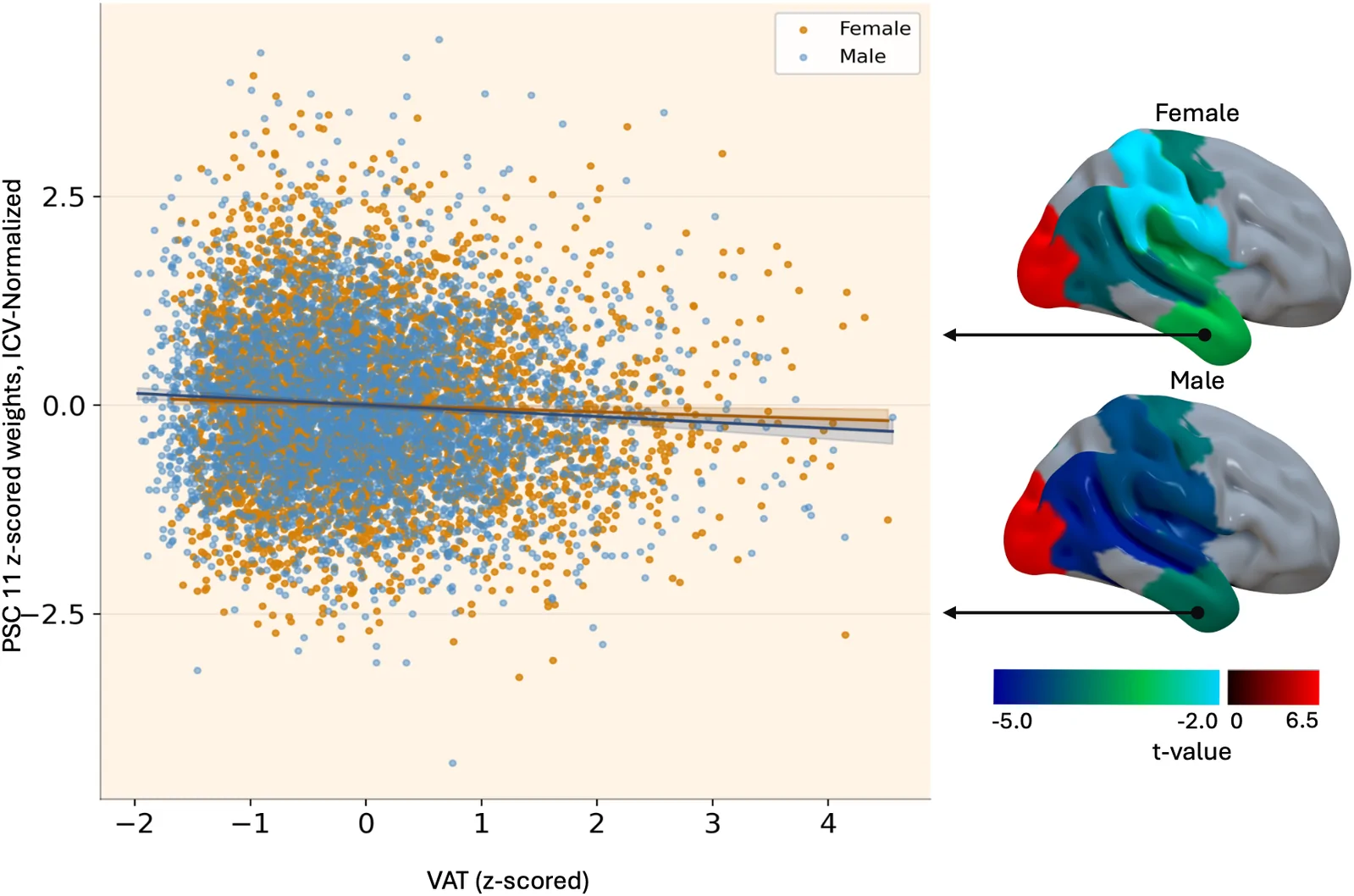
Association between visceral adipose tissue (VAT) and a representative cortical PSC (PSC 11). Points show individual participants (z-scored VAT vs. z-scored, ICV-normalized PSC loadings) with females and males plotted jointly; solid lines denote least-squares fits with 95% confidence bands. The brain surface rendering displays the t-statistics for the VAT association for all cortical PSCs that reached FDR significance, not significance of the PSCs themselves. For visualization, winner-takes-all maps were generated by assigning each voxel exclusively to the PSC with the highest loading value at that location. The resulting non-overlapping maps highlight the dominant spatial extent of each PSC, with colors representing the corresponding PSC-level t-statistic.

Across the 20 PSCs derived with csopNMF, higher visceral adipose tissue (VAT; UKB field 22407) was associated with altered GM integrity within 13 PSCs in females and 11 PSCs in males ($p_{\text{FDR}}\;<0.05$ ). Lower GM volume was associated with higher VAT (Fig. 8, Supplementary Figs. S1-S3) in parietal perirolandic and supramarginal territories (PSCs 5–6), the temporoparietal junction and superior temporal cortex (PSC 7), and temporal regions encompassing inferior temporal cortex and the temporal pole (PSCs 10–11). Figure 9 presents surface maps of t-statistics for all FDR-significant cortical components and a representative temporal-pole scatter relating z-scored VAT to z-scored, ICV-normalized loadings. Complete regression coefficients, 95% confidence intervals, t-statistics, p-values, and model-fit indices are reported in Supplementary Table S1; regression plots for all PSCs are provided in Supplementary Material S3 Figures S3–S5.

#### 3.2.3 Higher rank parcellations reveal hierarchy

**Fig. 10.**
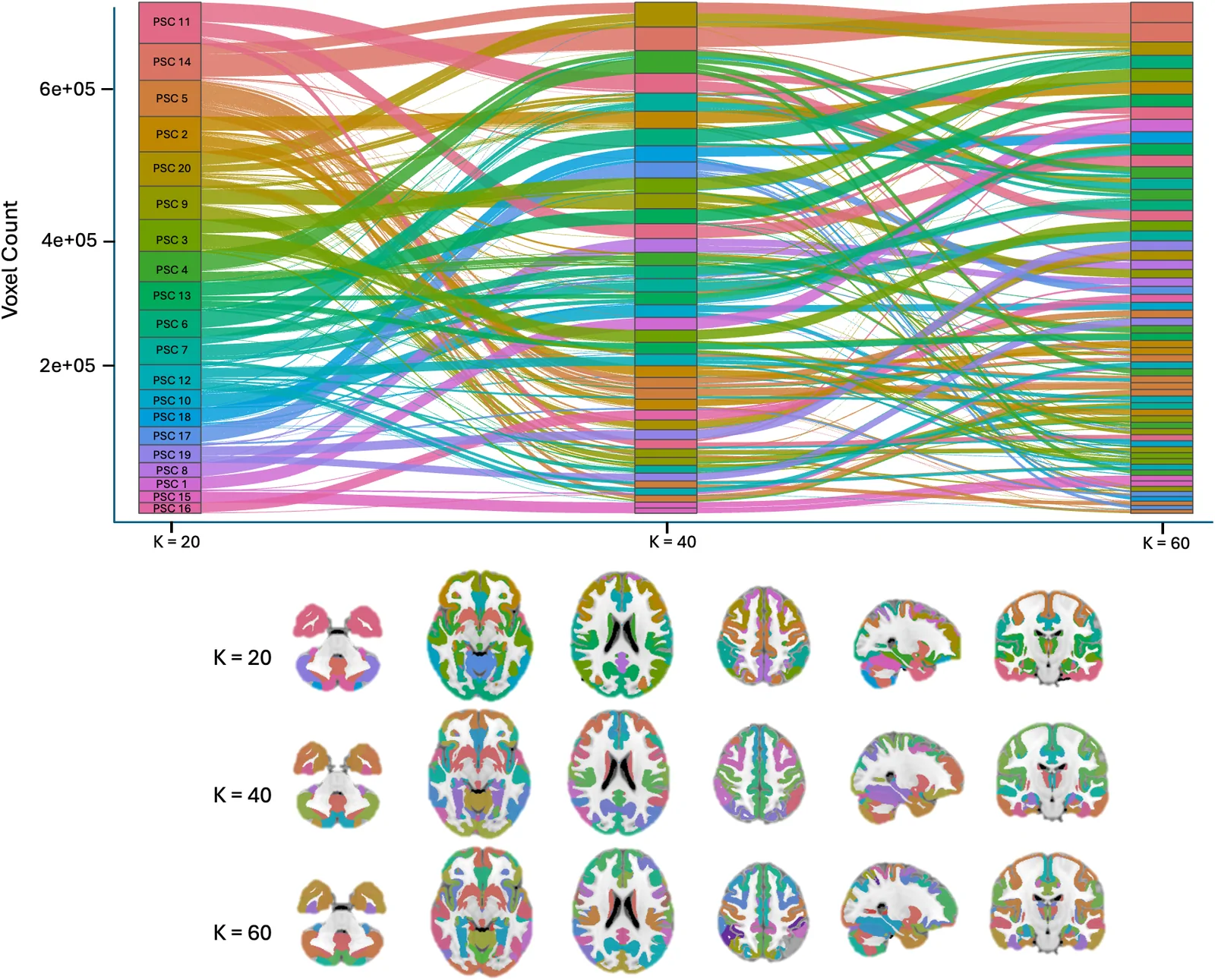
Hierarchical organization of PSCs across csopNMF model orders. Tracking voxel-level winner-takes-all assignments across rank-20, rank-40, and rank-60 csopNMF decompositions reveals a nested hierarchical organization in which broad PSCs progressively subdivide into finer-grained components at higher ranks (top). Increasing hemispheric lateralization is also observed in several cortical and cerebellar systems with increasing model order. Composite visualizations were generated using a winner-takes-all approach, in which each voxel was assigned to the PSC with the highest loading value across all components within a given decomposition. Representative views of rank-20, rank-40, and rank-60 decompositions are shown (bottom).

Next, we examined csopNMF solutions at higher model orders of 40 and 60 to assess the extent to which finer-scale PSCs were nested within the coarser rank-20 solution. To do so, we tracked voxel memberships across ranks and quantified the spatial overlap of PSCs as model order increased (Fig. 10). The higher-rank decompositions revealed a clear hierarchical organization, in which broad PSCs progressively subdivided into finer-grained patterns, as well as increasing hemispheric lateralization in several cortical systems (see Supplementary Material S4, Figs. S6 and S7).

## 4 Discussion

### 4.1 Methodological comparison with opNMF

In this study, we introduced csopNMF, an algorithm designed for large-scale neuroimaging that addresses the computational limits of opNMF. Using stochastic optimization and random compression, csopNMF scales efficiently to very large datasets while achieving approximation error and sparsity comparable with opNMF and preserving interpretability of components. We also integrated diverse sampling via DPP to reduce variance in stochastic updates and enable informed mini-batches. Because opNMF is non-convex with multiple local minima, we used NNDSVD initialization (Boutsidis & Gallopoulos, 2008) to stabilize solutions and expedite convergence. Across configurations, the resulting components reconstructed the data well and aligned with those from standard opNMF.

Beyond reconstruction accuracy, the proposed variants maintained spatial sparsity at the component level, yielding components with the same degree of anatomical localization observed in opNMF. We further quantified reproducibility using ARI and component-wise inner products and observed high agreement with opNMF across all configurations. To evaluate downstream utility, we used the loading coefficients in a brain-age regression task and found that prediction accuracy (as quantified by MAE and $R^{2}$) was indistinguishable from that obtained with opNMF loadings.

More broadly, NMF-based methods assume non-negative input data and are thus most naturally suited to modalities in which this assumption holds. Extending csopNMF to mixed-sign data such as those that often arise from processing functional MRI would require either mixed-sign factorization variants, such as Semi-NMF, or preprocessing strategies to enforce non-negativity (Ding et al., 2010; Fürböck, 2020; Ghanbari et al., 2017). For example, rescaling voxel-wise time courses by shifting them into the positive domain has previously been used successfully in the context of NMF (Cui et al., 2020; H. Li et al., 2017) and could, in principle, be adapted for csopNMF as well.

### 4.2 Application to UK Biobank and associations with VAT

After validation, we applied csopNMF to 10,000 UK Biobank participants and derived a 20-PSC solution. The resulting patterns delineated cortical, subcortical, and cerebellar PSCs with minimal overlap, preserving the spatial specificity characteristic of opNMF. We then examined associations of the 20 PSCs and VAT. Higher VAT was associated with altered GM integrity in 13 components in females and 11 components in males, with predominantly negative associations across cortical patterns and the cerebellum.

The negative temporal pole association aligns with population evidence linking adiposity to lower gray matter measures on MRI (Dekkers et al., 2019; García-García et al., 2019; Opel et al., 2021; Raji et al., 2024). The temporal pole is strongly implicated in higher-order memory and semantic processing (Patterson et al., 2007; Ralph et al., 2017), and it is among the early cortical territories to show atrophy along the Alzheimer’s disease continuum (Braak & Braak, 1991; Jack et al., 2018). Thus, the observed negative VAT–temporal-pole association is notable in the context of epidemiological evidence that adiposity is a modifiable midlife risk factor for late-life dementia (Livingston et al., 2020). Linking a body-composition phenotype to structural variation in a region with known vulnerability to neurodegenerative processes provides a plausible neurobiological pathway through which metabolic burden may influence dementia risk.

Cerebellar patterns showed a similar trend: of six cerebellar PSCs, all were significant in females and five were significant in males (Supplementary Table S1). The subcortical PSC (PSC 20) was significant in females but not significant in males, indicating a sex-dependent association with subcortical gray matter, consistent with prior reports (Dekkers et al., 2019). A limitation of this analysis is that the UK Biobank sample consisted of individuals without ICD-10 diagnoses at baseline. As a result, disease-related effects on brain structure may have been attenuated, potentially contributing to differences in the observed directionality of VAT associations.

### 4.3 Higher rank decompositions and hierarchical organization of structural covariance patterns in the UKB

To illustrate the behavior of the method beyond the rank 20 application, we also examined higher-rank decompositions at 40 and 60 components. The higher-rank decompositions suggest that csopNMF preserves the broad structural organization identified at lower model orders while progressively refining it into more specific PSCs as rank increases. The resulting patterns indicate a hierarchical organization, in which coarse components subdivide into finer subnetworks and, in some cases, become increasingly lateralized. Although these observations are presented here as illustrative examples rather than a comprehensive neurobiological characterization, they highlight the ability of csopNMF to recover structural covariance patterns across multiple levels of granularity in large-scale neuroimaging data.

A limitation of the present study is that, although the UK Biobank imaging protocol is highly standardized across sites, we did not explicitly apply retrospective harmonization procedures or model head motion effects in the downstream analysis. The use of identical scanner hardware, matched software versions, and a fixed acquisition protocol in UKB reduces site-related variability relative to more heterogeneous multicenter datasets; however, some residual scanner- or site-related variance may still remain. Established harmonization methods have been shown to reduce such variance while preserving biological signal (Pomponio et al., 2020; Zhou et al., 2023), and these approaches are fully compatible with the present framework. Future applications of csopNMF to less standardized datasets, or to pooled multisite cohorts, may, therefore, benefit from incorporating harmonization methods, motion-related quality control measures, and explicit nuisance modeling to further reduce non-biological sources of variance.

### 4.4 On csopNMF parameters: Mini-batch, compression, and rank

In practice, the choice of csopNMF parameters should be guided by balancing computational resources. For the mini-batch size p, our results, theoratical analysis, and prior work (Bani et al., 2023) indicate that csopNMF is robust across a range of mini-batch configurations. In the present study, five independent runs using the selected mini-batch size in the UKB produced highly consistent solutions based on ARI, mean, and median inner products, further demonstrating the robustness of the selected configuration to stochastic mini-batch sampling. We, therefore, recommend the end user to select a mini-batch size that is comfortably larger than their target rank and comfortably fits in their memory. In our experiments, we found a mini-batch size of 125 to yield sufficient approximation of the opNMF solution.

For compression, the goal is similar to balance efficiency against fidelity to the original opNMF solution. Our experiments show that csopNMF remains robust across a range of compression settings, including relatively aggressive compression, with only modest effects on approximation error and component similarity. Theoretically, increasing the oversampling parameter $k_{ov}$ improves the quality of the randomized approximation and reduces compression error, while relatively small values are often sufficient in practice. Following established guidance from randomized linear algebra (Halko et al., 2011), small oversampling values such as 5, 10, or maximum 2×k generally provide strong performance, making them a reasonable starting point for practical applications. Taken together, these observations suggest that csopNMF parameters need not be tuned to a single optimal setting; rather, they should be chosen to accommodate available computational resources while maintaining stable and reproducible factorization results.

Rank selection remains an important consideration in csopNMF, as in NMF-based methods more broadly. Although the stochastic optimization and compression steps may introduce some additional variability relative to full opNMF, csopNMF should still follow standard framework for identifying an appropriate number of factors. In practice, the most informative strategy remains reproducibility-based screening across candidate model orders, particularly through split-half analyses (Nazeri et al., 2022), in which the data are decomposed separately across a range of ranks and the resulting solutions are compared using measures such as ARI or component similarity. Reconstruction error provides an important complementary criterion, especially when considered together with its rate of change as model order increases. In the present study, our primary objective was not to perform a systematic rank-selection analysis, but rather to establish csopNMF as a scalable approximation to opNMF.

### 4.5 On mini-batch sampling strategies

An additional consideration in csopNMF is the choice of mini-batch sampling strategy. In principle, mini-batch construction need not be limited to uniform random sampling, and could instead be informed by covariates of the data or imaging features. This motivation is closely related to our use of mini-batch diversification, since diversified sampling can improve the representativeness of the selected subsets at the population level and, from an optimization perspective, reduce the variance of stochastic updates and accelerate error decay. In the present study, we incorporated DPP-based diversification strategy. However, because both OASIS and UK Biobank are relatively homogeneous datasets composed largely of healthy individuals, we did not observe a clear advantage of DPP-based sampling over standard stochastic sampling in the results. Moreover, DPP sampling incurs additional computational cost through the construction of the sampling kernel. Taken together, these observations suggest that while uniform stochastic sampling may be sufficient in relatively homogeneous cohorts, alternative sampling strategies, including DPP-based or more explicitly relevance-informed probabilistic approaches, may be especially useful in more heterogeneous datasets or in applications where certain subjects or features are of biological importance. Future work will focus on applying the algorithm at various ranks to explore brain structure and organization in health and disease in large-scale diverse neuroimaging studies.

### 4.6 Conclusions

Taken together, these findings establish csopNMF as a scalable and interpretable framework for population-scale neuroimaging that not only recovers biologically meaningful patterns of structural covariance and relates them to relevant biological phenotypes, but also overcomes a central computational barrier that has limited the application of opNMF in large cohorts. Beyond empirical performance, csopNMF is supported by a theoretical analysis guaranteeing convergence to the opNMF solution under stochastic and compressed updates. A further advantage is that, unlike compression frameworks that operate on the full data matrix, our approach compresses mini-batches, which avoids the memory burden of storing a full projection basis when both sample size and feature dimension are large, and eliminates reliance on distributed QR decompositions (e.g., MapReduce-based TSQR). This design allows csopNMF to retain solution fidelity while enabling deployment on standard computing environments, thereby extending the practical usability of orthonormal projective NMF in big-neuroimaging applications.

## Supplementary Material

Supplementary Material

## Data Availability

The code for this project is publicly available at https://github.com/sotiraslab/csopNMF. The OASIS dataset used in this work is publicly available from the OASIS Brains repository. Access requires user registration and acceptance of the OASIS data use terms. All preprocessing steps and analysis parameters are described in the paper, and any additional details can be provided upon reasonable request. UK Biobank data are available to bona fide researchers by application to UK Biobank and approval of a research proposal. This study used data accessed under application number 47267. In accordance with UK Biobank’s material transfer and data access terms, we cannot publicly share the raw UKB data. Aggregated results and analysis are provided in the paper (and further details can be shared upon reasonable request) to enable reproducibility.
