# Supplementary material for: Taming dimensionality in big neuroimaging data: Efficient orthonormal projective NMF via stochastic learning and data compression
Source: Imaging Neurosci (Camb). 2026 Sep 8;4:IMAG.a.1355. doi: 10.1162/IMAG.a.1355 (PMC13556794; doi:10.1162/IMAG.a.1355)
Supplement: Supplementary file 1 [file IMAG.a.1355_supp.pdf]

S1 Supplementary Material: csopNMF Predicted Brain-age Gap and Cognitive Associations

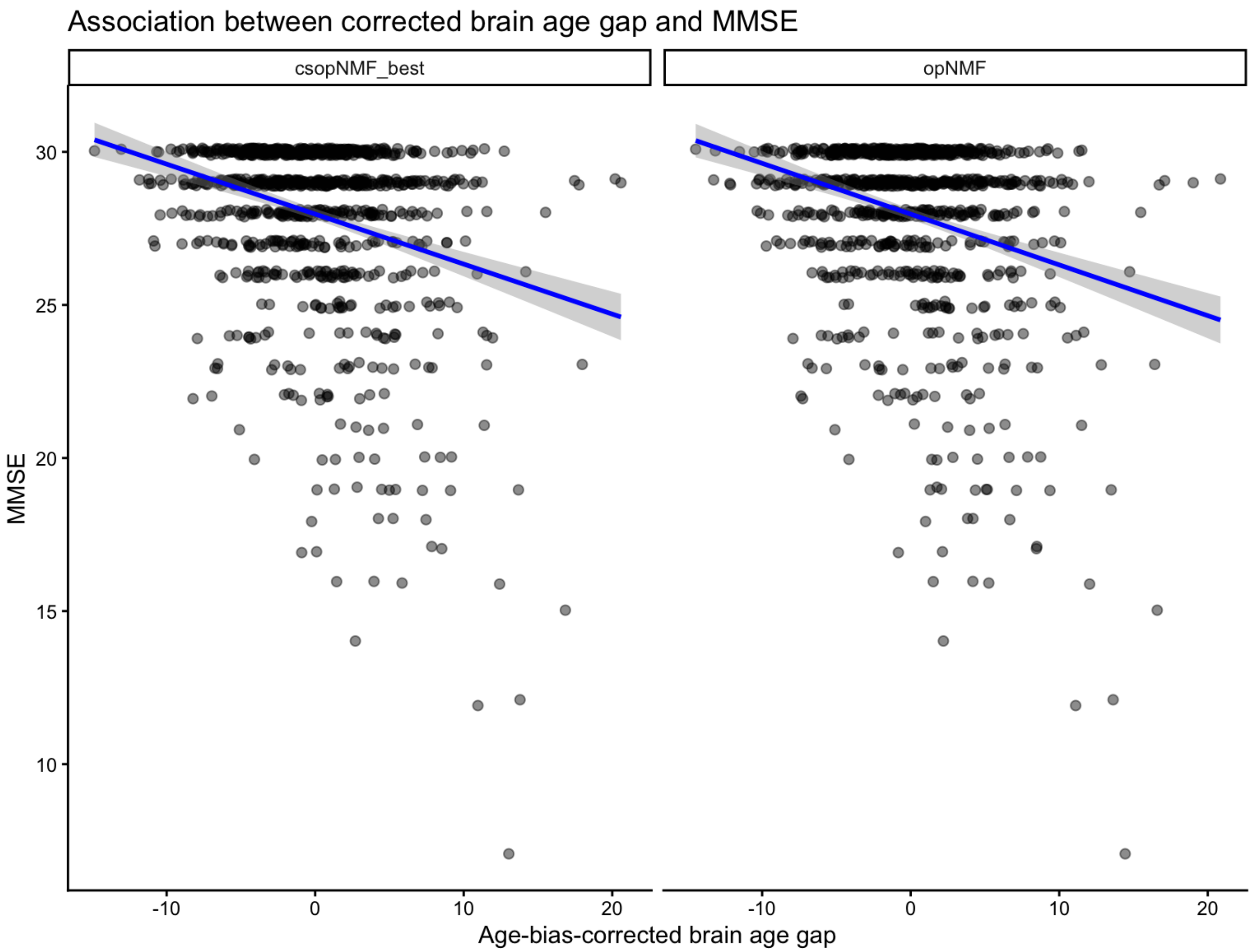

Figure S1: Scatter plots showing relationship between brain age gap and MMSE for opNMF and csopNMF-derived loading coefficients. Each point represents one subject, with jitter added for visualization as MMSE is highly concentrated. Briefly, chronological age was predicted from the component loading coefficients using repeated 10-fold cross-validation. For each subject, we computed predicted age and brain age gap (BAG), defined as predicted age minus chronological age. We also computed an age-bias-corrected BAG by residualizing BAG with respect to chronological age within each model. These subject-level measures were then matched to corresponding MMSE scores. We evaluated the association between age-bias-corrected BAG and MMSE using Spearman correlation. We observed a statistically significant negative association between corrected BAG and MMSE for both approaches. The fitted line shows the trend with 95% confidence interval. csopNMF (r=-0.197, p-value < 0.001) and opNMF (r=-0.194, p-value < 0.001).

S2 Supplementary Material: Mini-batch size Stability Analysis

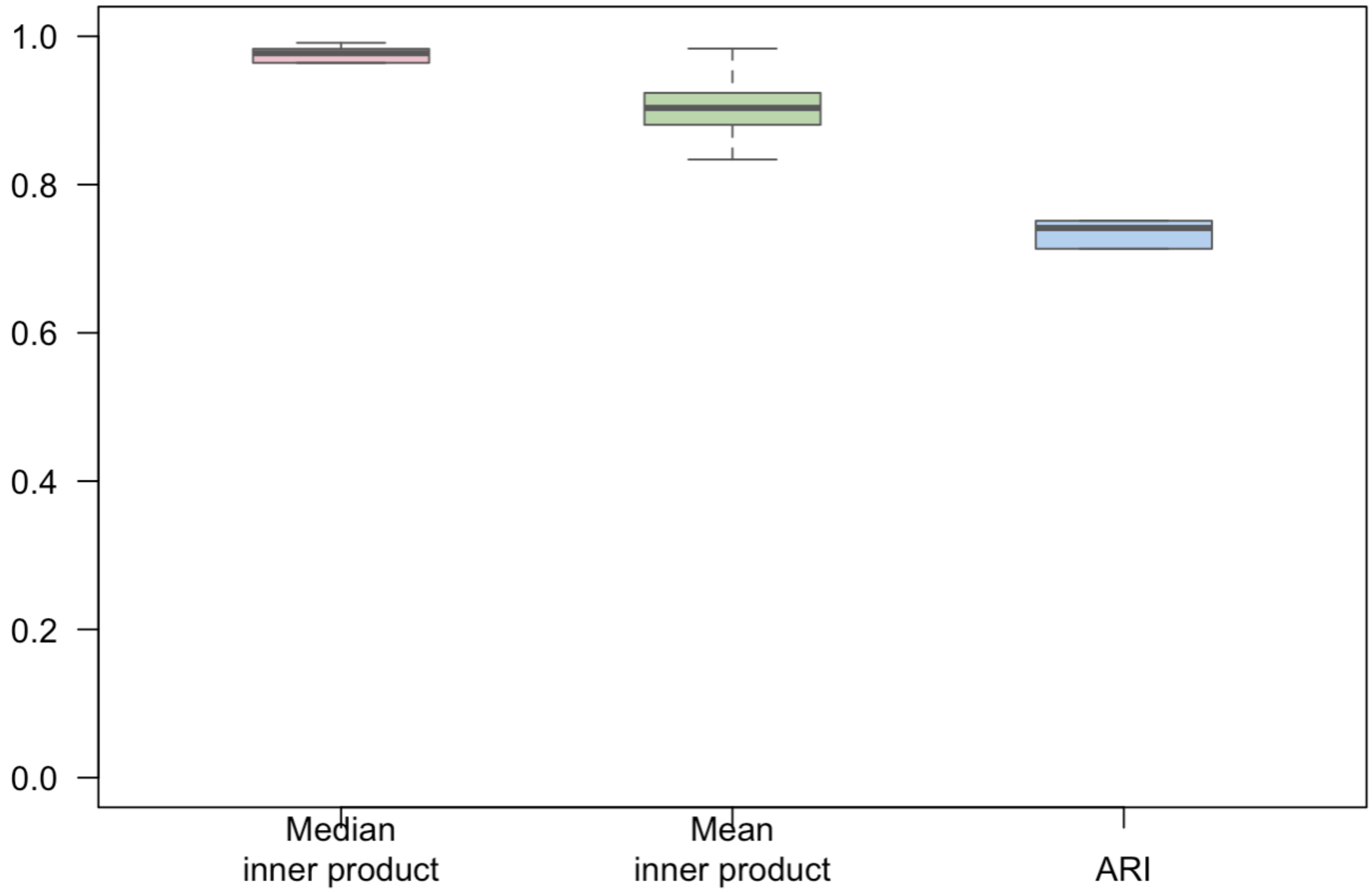

Figure S2: Reproducibility across five different independent runs on the selected batch size on the UKB. Runs were assessed under randomized sampling where each run used a different random seed. The boxplots summarize the component agreement across runs for the median inner product (0.97 ± 0.02), mean inner product (0.91 ± 0.05), and ARI (0.75 ± 0.06).

S3 Supplementary Material: Regression Results

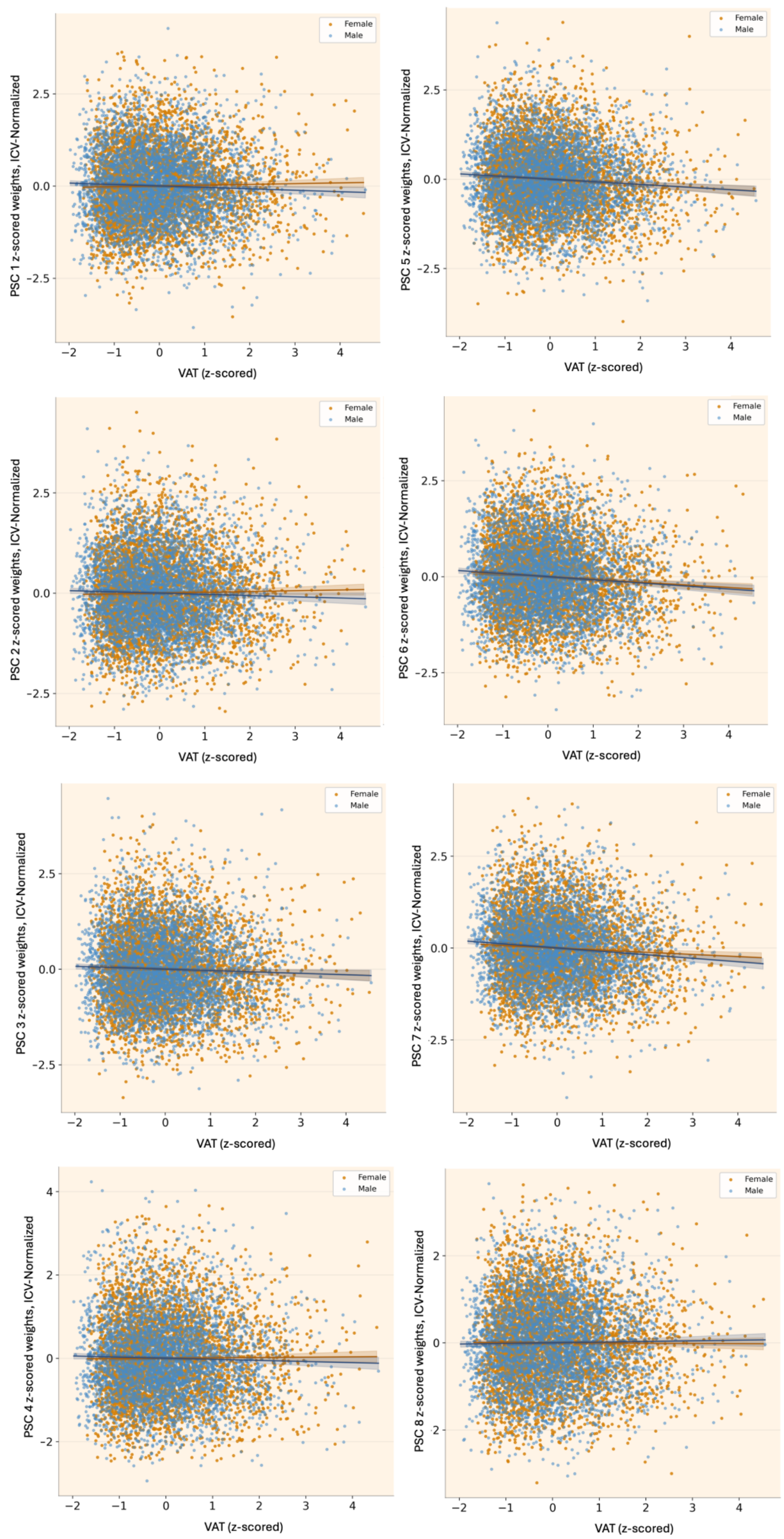

Figure S3: Scatter plots of z-scored visceral adipose tissue (VAT) versus z-scored, ICV-normalized PSC loadings for frontal and parietal PSCs (1-8), with females and males plotted on the same axes. Points show individuals; solid lines show least-squares fits with 95% confidence bands.

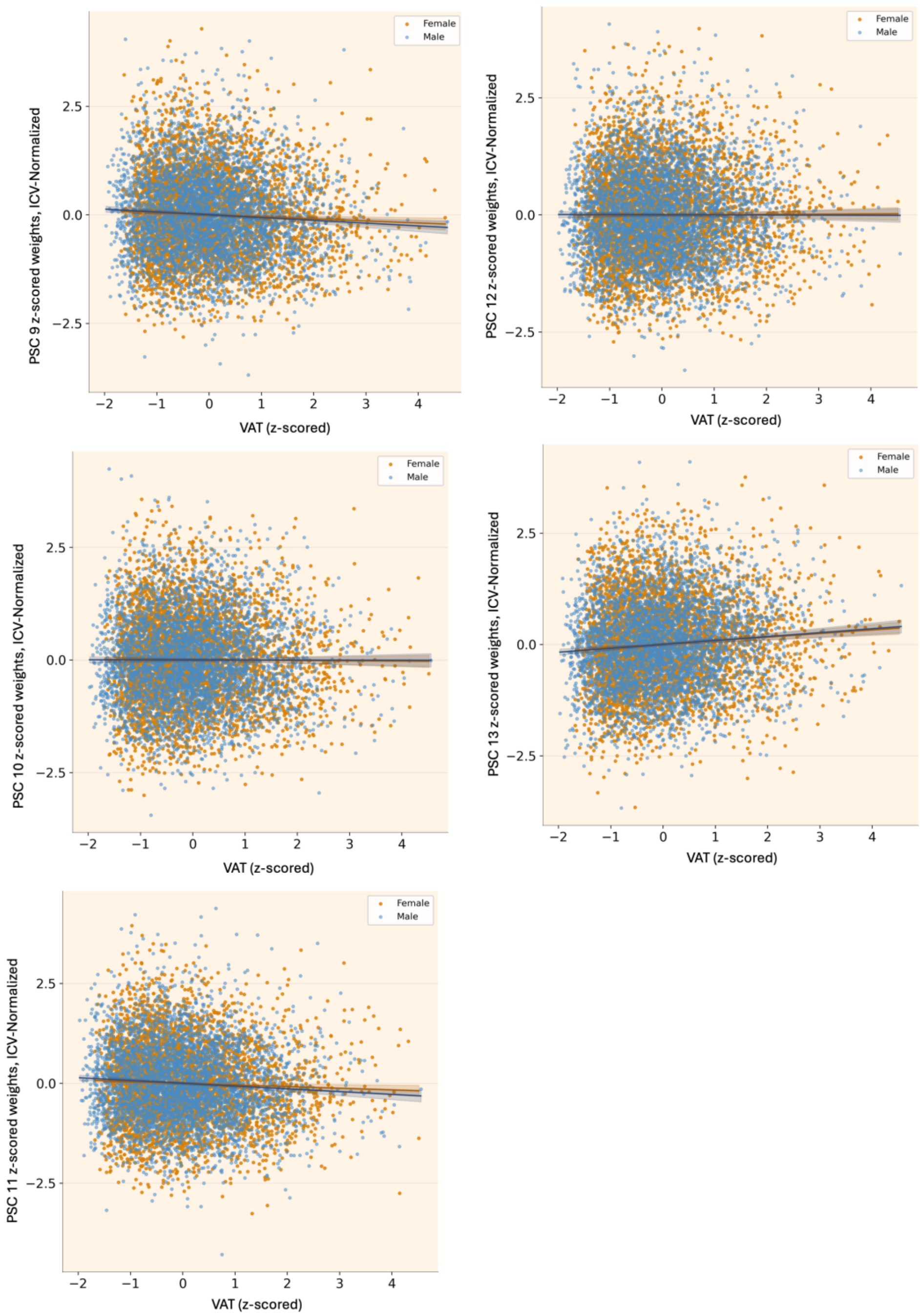

Figure S4: Scatter plots of z-scored visceral adipose tissue (VAT) versus z-scored, ICV-normalized PSC loadings for temporal, cingulate, and occipital PSCs (9-13), with females and males plotted on the same axes. Points show individuals; solid lines show least-squares fits with 9

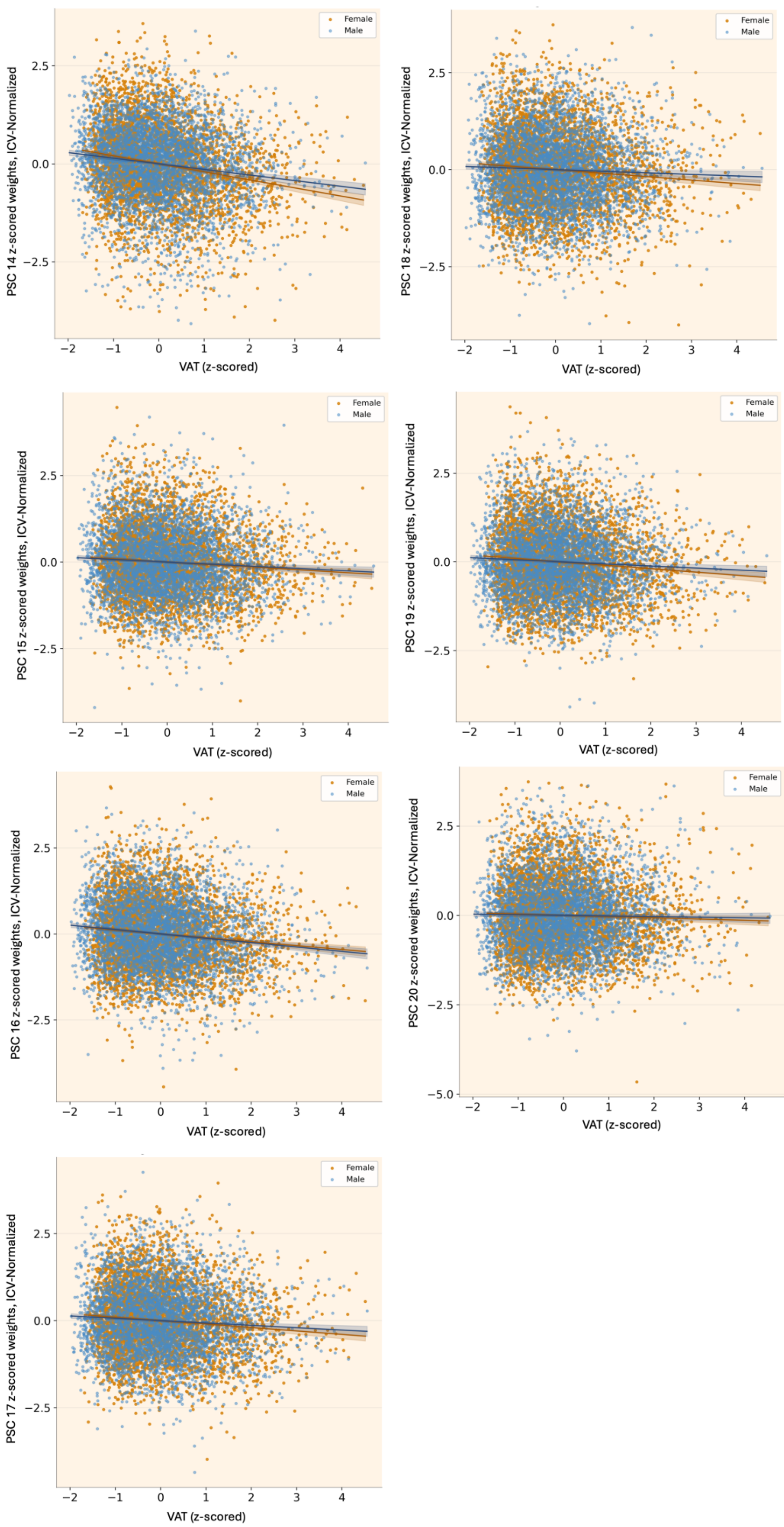

Figure S5: Scatter plots of z-scored visceral adipose tissue (VAT) versus z-scored, ICV-normalized PSC loadings for subcortical and cerebellar PSCs (14-20), with females and males plotted on the same axes. Points show individuals; solid lines show least-squares fits with 95% confidence bands.

Table S1: Adjusted regression results for visceral adipose tissue and patterns of structural covariance loadings

	Female (n=4374)						Male (n=3886)					
**PSC**	β_{VAT}	95% CI		p_{VAT}	t_{VAT}	FDR	VAT_{β}	95% CI		p_{VAT}	t_{VAT}	FDR
1	4.72E-08	-8.54E-08	1.80E-07	0.485	0.698		-1.33E-07	-2.66E-07	-3.43E-11	0.0499	-1.96	
2	8.97E-08	-5.48E-08	2.34E-07	0.224	1.216		-8.83E-08	-2.33E-07	5.59E-08	0.230	-1.199	
3	-9.34E-08	-2.34E-07	4.78E-08	0.195	-1.296		-8.09E-08	-2.27E-07	6.55E-08	0.278	-1.082	
4	3.04E-08	-1.19E-07	1.80E-07	0.691	0.398		-6.79E-08	-6.79E-08	8.48E-08	0.383	-0.871	
5	-2.32E-07	-3.80E-07	-8.54E-08	0.002	-3.097	*	-2.39E-07	-3.85E-07	-9.32E-08	0.001	-3.211	*
6	-2.12E-07	-3.73E-07	-5.18E-08	0.009	-2.592	*	-2.94E-07	-4.54E-07	-1.35E-07	0.0003	-3.618	*
7	-2.24E-07	-3.83E-07	-6.49E-08	0.005	-2.758	*	-3.91E-07	-5.48E-07	-2.33E-07	1.11E-06	-4.871	*
8	2.88E-08	-1.26E-07	1.84E-07	0.716	0.364		1.57E-07	-4.77E-09	3.20E-07	0.057	1.902	
9	-2.03E-07	-3.72E-07	-3.36E-08	0.0188	-2.349	*	-2.59E-07	-4.28E-07	-9.09E-08	0.002	-3.017	*
10	-1.15E-07	-2.86E-07	5.47E-08	0.184	-1.330		1.24E-08	-1.65E-07	1.89E-07	0.891	0.137	
11	-3.40E-07	-5.44E-07	-1.37E-07	0.001	-3.275	*	-4.28E-07	-6.37E-07	-2.18E-07	6.20E-05	-4.005	*
12	1.60E-08	-1.21E-07	1.53E-07	0.819	0.228		1.48E-08	-1.22E-07	1.52E-07	0.832	0.212	
13	6.15E-07	4.18E-07	8.12E-07	8.92E-10	6.127	*	6.79E-07	4.83E-07	8.74E-07	9.57E-12	6.813	*
14	-1.59E-06	-1.82E-06	-1.36E-06	1.54E-42	-13.67	*	-1.08E-06	-1.32E-06	-8.31E-07	5.64E-18	-8.639	*
15	-5.42E-07	-7.21E-07	-3.63E-07	3.16E-09	-5.923	*	-3.05E-07	-4.84E-07	-1.26E-07	8.00E-04	-3.339	*
16	-4.77E-07	-6.60E-07	-2.95E-07	3.07E-07	-5.119	*	-3.40E-07	-5.32E-07	-1.48E-07	5.00E-04	-3.468	*
17	-3.59E-07	-5.10E-07	-2.08E-07	2.98E-06	-4.672	*	-9.92E-08	-2.50E-07	5.14E-08	0.196	-1.291	
18	-7.44E-07	-9.31E-07	-5.56E-07	8.20E-15	-7.764	*	-7.79E-07	-9.78E-07	-5.80E-07	1.71E-14	-7.671	*
19	-6.09E-07	-8.06E-07	-4.13E-07	1.32E-09	-6.065	*	-3.00E-07	-4.96E-07	-1.05E-07	0.002	-3.016	*
20	-2.79E-07	-4.08E-07	-1.49E-07	2.52E-05	-4.213	*	-8.13E-08	-2.09E-07	4.60E-08	0.210	-1.252	

S4 Supplementary Material: Higher Order Models and Hierarchy

Within the cortex, the frontal lobe was subdivided into 8 PSCs in the rank-40 solution (PSC 1–8) and 10 PSCs in the rank-60 solution (PSC 1–10) (Figure S6). For example, PSC 4 in the rank-20 solution, corresponding to medial frontal and orbitofrontal regions, subdivided into PSCs 7 and 8 at rank 40, which further refined into PSCs 9, 10, and 11 at rank 60. Similarly, PSC 2 in the rank-20 solution, corresponding to middle frontal cortex, split into PSCs 2 and 3 at rank 40 and further into PSCs 2–5 at rank 60 and 3 at rank 40 and into PSCs 2–5 at rank 60 (Figure S6).

The parietal lobe likewise showed both hierarchical subdivision and increasing lateralization. PSCs that were relatively symmetric at rank 20 became separated into more distinct left- and right-dominant PSCs at higher ranks (Figure S6). In total, the parietal lobe was represented by 11 PSCs in the rank-40 solution (PSC 9–19) and by 19 PSCs in the rank-60 solution (PSC 12–30) (Figure S6). The temporal lobe, which was summarized by 2 PSCs in the rank-20 solution, expanded to 3 PSCs at rank 40 (PSC 20–22) and 8 PSCs at rank 60 (PSC 31–38) (Figure S6). The occipital cortex, represented by PSC 13 at rank 20, subdivided into 3 PSCs at rank 40 (PSC 23–25) and 4 PSCs at rank 60 (PSC 39–42)(Figure S6). The cingulate gyrus, represented by PSC 12 at rank 20, was resolved into 2 PSCs at rank 40 (PSC 27 and 28) and 3 PSCs at rank 60 (PSC 43–45)(Figure S6). Finer regional specificity also emerged with increasing granularity; for instance, the pericalcarine cortex was distinctly isolated as PSC 26 in the rank-40 solution and PSC 46 in the rank-60 solution (Figure S6).

Similarly, finer subcortical organization emerged at higher model orders. In the rank-40 solution, the subcortical structures were resolved into 3 PSCs: PSC 38 captured the amygdala and hippocampus, whereas PSCs 39 and 40 captured the basal ganglia (Figure S7). In the rank-60 solution, the amygdala and hippocampus remained grouped within PSC 59, while the basal ganglia were further subdivided into 3 PSCs (PSCs 57, 58, and 60), indicating additional refinement of subcortical structure at higher rank (Figure S7).

The cerebellum likewise showed both hierarchical subdivision and increasing lateralization, particularly within the lateral hemispheric regions. In the rank-40 solution, the cerebellum was represented by 9 PSCs, with PSCs 29, 32, 33, and 34 covering medial and intermediate zones, and PSCs 30, 31, 35, 36, and 37 covering more lateral hemispheric regions (Figure S7). In the rank-60 solution, medial cerebellar structure was captured by PSCs 47, 48, 54, 55, and 56, whereas lateral hemispheric regions were represented by PSCs 49, 50, 51, 52, and 53 (Figure S7).

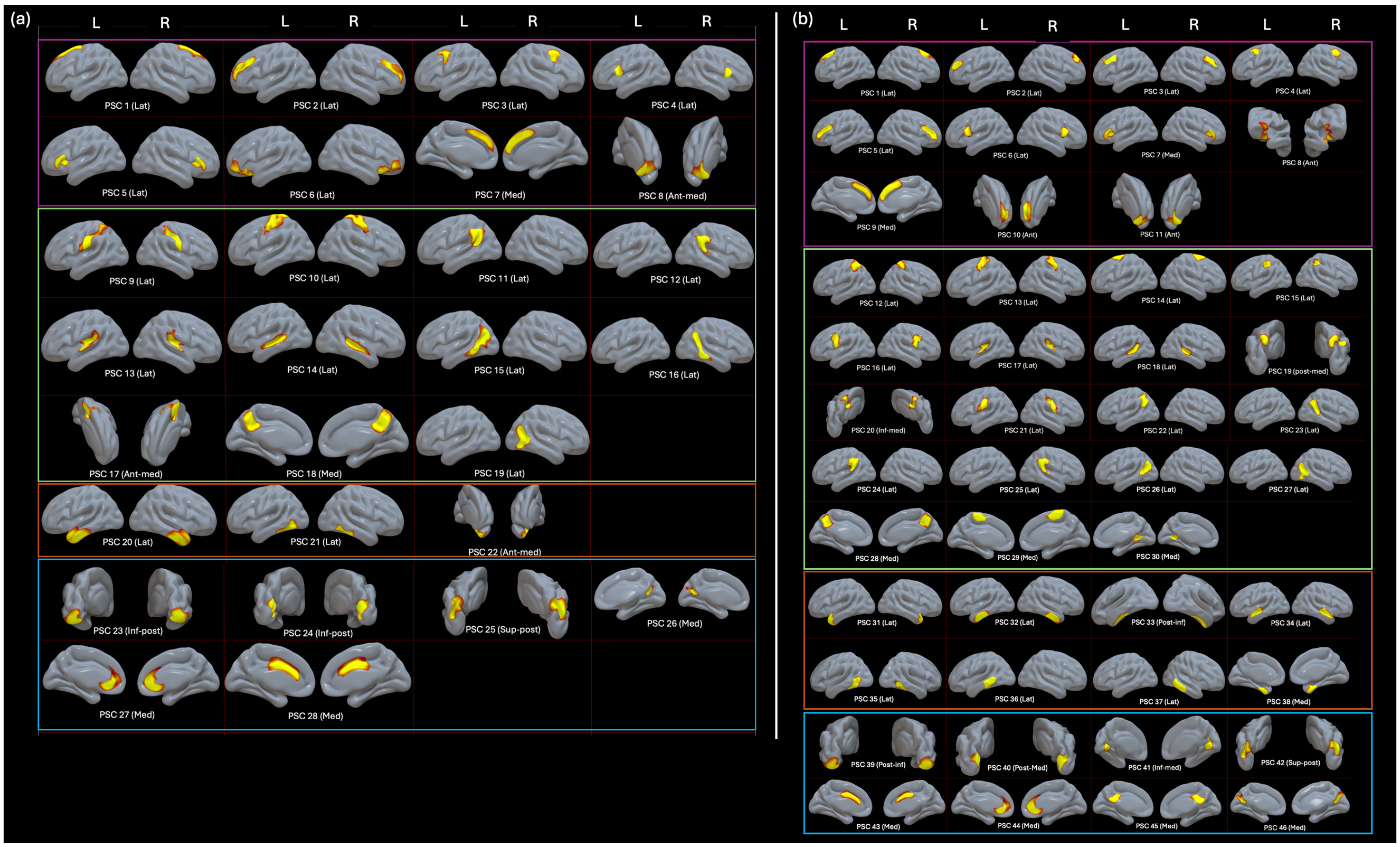

Figure S6: Cortical PSCs from higher-rank csopNMF decompositions in the UK Biobank sample. (a) Rank-40 solution; (b) Rank-60 solution. Compared with the rank-20 decomposition, higher-rank solutions reveal hierarchical subdivision of broad cortical PSCs into finer frontal (magenta box), parietal (green box), temporal (orange box), occipital and cingulate patterns (blue box), together with increasing hemispheric lateralization in several systems.

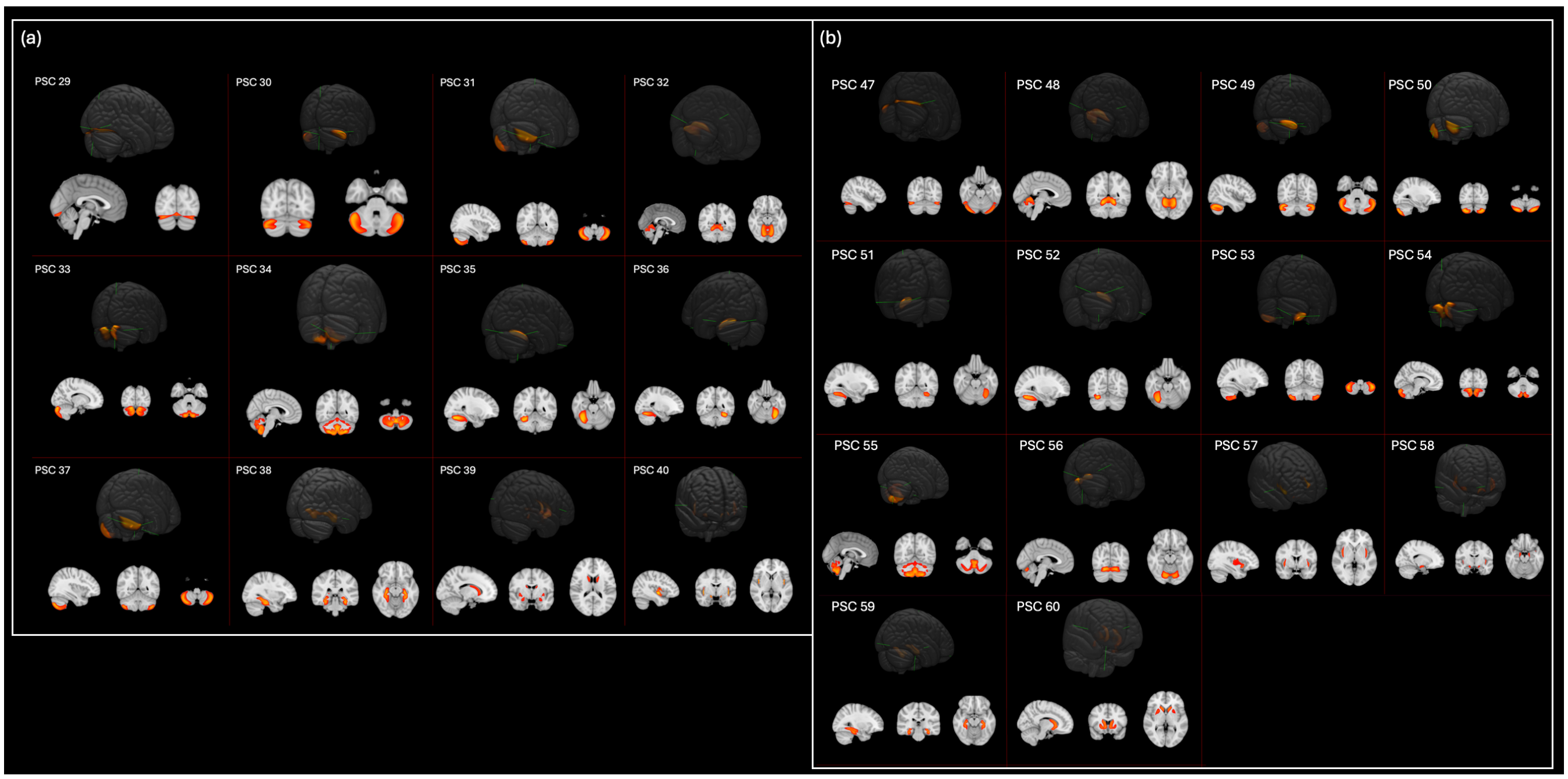

Figure S7: Cerebellar and subcortical PSCs from higher-rank csopNMF decompositions in the UK Biobank sample. (a) Rank-40 solution; (b) Rank-60 solution. Higher-rank decompositions reveal finer differentiation of subcortical structures, including separation of amygdalar-hippocampal and basal ganglia patterns, as well as hierarchical and lateralized refinement of cerebellar medial, intermediate, and lateral hemispheric regions.
